# A genome alignment of 120 mammals highlights ultraconserved element variability and placenta-associated enhancers

**DOI:** 10.1093/gigascience/giz159

**Published:** 2020-01-03

**Authors:** Nikolai Hecker, Michael Hiller

**Affiliations:** 1 Max Planck Institute of Molecular Cell Biology and Genetics, Pfotenhauerstr. 108, 01307 Dresden, Germany; 2 Max Planck Institute for the Physics of Complex Systems, Noethnitzer Str. 38, 01187 Dresden, Germany; 3 Center for Systems Biology Dresden, Pfotenhauerstr. 108, 01307 Dresden, Germany

**Keywords:** genome alignment, comparative gene annotation, ultraconserved elements, enhancers, mammals

## Abstract

**Background:**

Multiple alignments of mammalian genomes have been the basis of many comparative genomic studies aiming at annotating genes, detecting regions under evolutionary constraint, and studying genome evolution. A key factor that affects the power of comparative analyses is the number of species included in a genome alignment.

**Results:**

To utilize the increased number of sequenced genomes and to provide an accessible resource for genomic studies, we generated a mammalian genome alignment comprising 120 species. We used this alignment and the CESAR method to provide protein-coding gene annotations for 119 non-human mammals. Furthermore, we illustrate the utility of this alignment by 2 exemplary analyses. First, we quantified how variable ultraconserved elements (UCEs) are among placental mammals. Leveraging the high taxonomic coverage in our alignment, we estimate that UCEs contain on average 4.7%–15.6% variable alignment columns. Furthermore, we show that the center regions of UCEs are generally most constrained. Second, we identified enhancer sequences that are only conserved in placental mammals. We found that these enhancers are significantly associated with placenta-related genes, suggesting that some of these enhancers may be involved in the evolution of placental mammal-specific aspects of the placenta.

**Conclusion:**

The 120-mammal alignment and all other data are available for analysis and visualization in a genome browser at https://genome-public.pks.mpg.de/and for download at https://bds.mpi-cbg.de/hillerlab/120MammalAlignment/.

## Introduction

Comparative genomics has substantially contributed to detecting and classifying functional regions in genomes and understanding genome evolution [1, 2]. A foundation for most comparative genomics analyses are alignments between entire genomes. Several computational methods rely on genome alignments for annotating coding and non-coding genes, and genome alignments have been used to detect novel coding exons, revise exon-intron boundaries, and correct the positions of annotated start or stop codons [3–9]. Many gene or exon finders utilize genome alignments to increase the reliability of their predictions [10–14]. In addition, genome alignments provide an effective way to project genes from a reference species annotation to aligned (query) species [15–17]. Genome alignments have also been used to identify regions that evolve under purifying selection and thus likely have a biological function [18, 19]. Approximately 3–15% of the human genome is estimated to be evolutionarily constrained [20], and most of the constraint detected in genome alignments is located in conserved non-exonic elements that often overlap *cis*-regulatory elements such as enhancers [21, 22]. Furthermore, genome alignments have been instrumental for understanding the evolution of genomes, which uncovered genomic determinants of trait differences [23–30] and provided insights into evolutionary history and species’ biology [31–34].

A key factor affecting the power of comparative analyses is the number of species included in the genome alignment. Because higher taxonomic coverage increases the power to detect evolutionary constraint [35] and yields more robust results in phylogenetic and evolutionary studies [36, 37], it is desirable to include many sequenced genomes to capture the diversity of species in a respective clade. While the availability of sequenced genomes was a limiting factor in the past, advances in sequencing and assembly technology have led to a wealth of sequenced genomes, illustrated by the availability of >100 mammalian genomes.

To provide a comparative genomics resource that reflects the increased availability of sequenced mammals and is easily accessible to genomics experts and non-experts, we generated a multiple genome alignment of 120 mammals. We used the human gene annotation and Coding Exon-Structure Aware Realigner (CESAR) to provide comparative gene annotations for all 119 non-human mammals. Furthermore, we demonstrate the utility of the high species coverage in our alignment by (i) quantifying how variable ultraconserved elements are among placental mammals and (ii) identifying *cis*-regulatory elements (enhancers) that arose in the placental mammal lineage and showing that these enhancers are significantly associated with placenta-related genes. To facilitate comparative analyses using our resources, we provide the multiple genome alignment, a phylogenetic tree, conserved regions including GERP++ and PhastCons conservation scores, and the comparative gene annotations in a UCSC genome browser installation [38].

## Results and Discussion

### Generating a multiple genome alignment of 120 mammals

**Figure 1:**
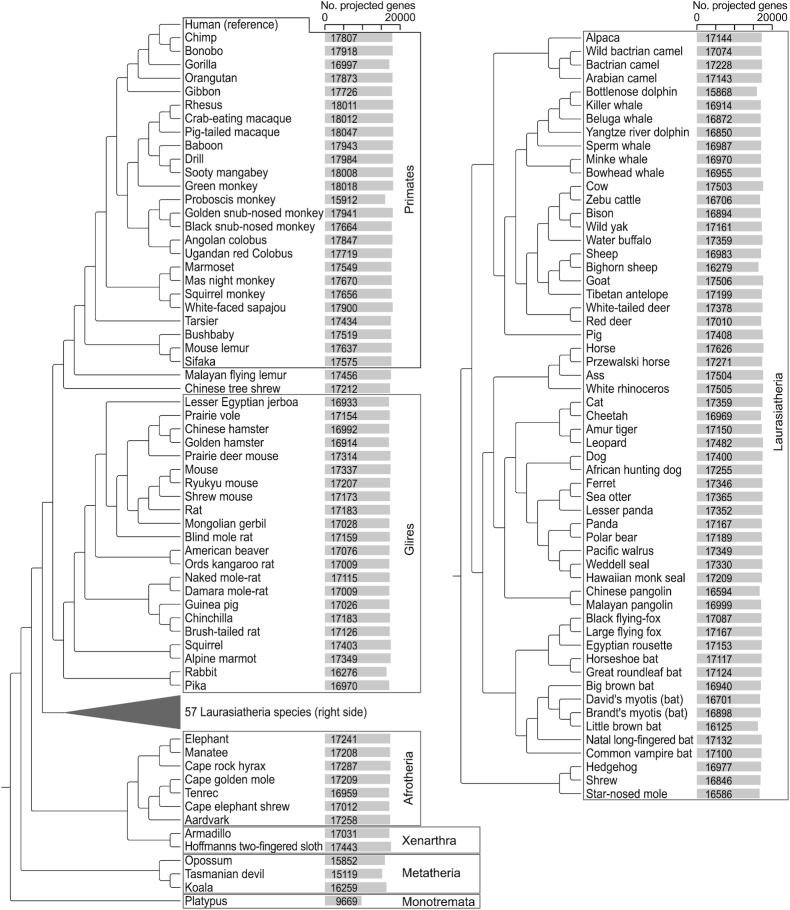
Phylogeny of 120 mammals included in our alignment and number of annotated genes. Bars visualize the number of human genes for which we projected ≥1 intact exon. Major groups of mammals are indicated. The 57 Laurasiatheria species are shown on the right side for space reasons.

To compute a comprehensive multiple genome alignment of mammals, we used human as the reference species and aligned 119 non-human mammals that have genome assemblies with a scaffold N50 value of ≥100,000 (Supplementary Table 1). The phylogeny of these 120 species is shown in Fig. 1. The workflow and methods used to compute the alignment are shown in Supplementary Fig. 1.

### Comparative gene and conserved element annotation

We first used our alignment to annotate protein-coding genes in all 119 non-human mammals. To this end, we used CESAR [15, 39, 40] to project all coding exons of human genes and annotated intact exons in all 119 non-human aligned mammals. Intact exons are defined as having an intact open reading frame without premature stop codons, and 2 consensus splice sites (internal exons) or 1 consensus splice site and a start (first exon) or stop (last codon) codon. Because intact exons can be missing owing to assembly gaps and assembly base errors [32, 34, 41, 42], we determined for each species the number of genes where ≥1 intact exon was annotated. We found that between 15,868 and 18,047 of the human genes have ≥1 intact exon alignment in placental mammals (Fig. 1). For marsupials, we annotated between 15,119 and 16,259 genes. In the platypus, a member of the monotremes, we annotated 9,669 genes (Fig. 1).

Second, in addition to annotating protein-coding genes, we annotated genomic regions that likely evolve under evolutionary constraint (purifying selection). To this end, we used PhastCons [18], a phylogenetic hidden Markov model method, and GERP++ [43], a method that directly measures the number of substitutions per site that were rejected by purifying selection. We applied both methods to detect regions constrained across all mammals in our alignment. PhastCons and GERP++ identified 13,257,408 and 1,612,714 conserved elements covering 5.5% and 9.9% of the human genome, respectively.

### Case study 1: Quantifying divergence in ultraconserved elements

The large number of mammalian species in our genome alignment provides an opportunity to quantify how variable highly conserved genomic elements are across placental mammals. We focused on a subset of highly conserved elements, called ultraconserved elements (UCEs), that have attracted much attention because deletions of several of these elements does not affect cellular fitness and resulted in viable organisms [44–46]. UCEs were originally defined as genomic regions that are ≥200 bp long (the largest UCE is 779 bp long) and have identical sequences between human, mouse, and rat [47]. Despite the fact that only 3 mammals were used to identify these genomic regions, UCEs are also highly conserved in other mammals and typically align to non-mammalian vertebrates [48]. For example, human UCE sequences align to chicken with a mean sequence identity of 96% [47]. Transgenic enhancer assays have shown that many non-exonic UCEs overlap regulatory elements that drive gene expression during development [22], and a recent study showed that ultraconserved enhancers are required for normal development in mice [45]. UCEs are not mutational cold spots because there is genetic variation in the human population; however, derived mutations are under strong purifying selection [49].

**Figure 2:**
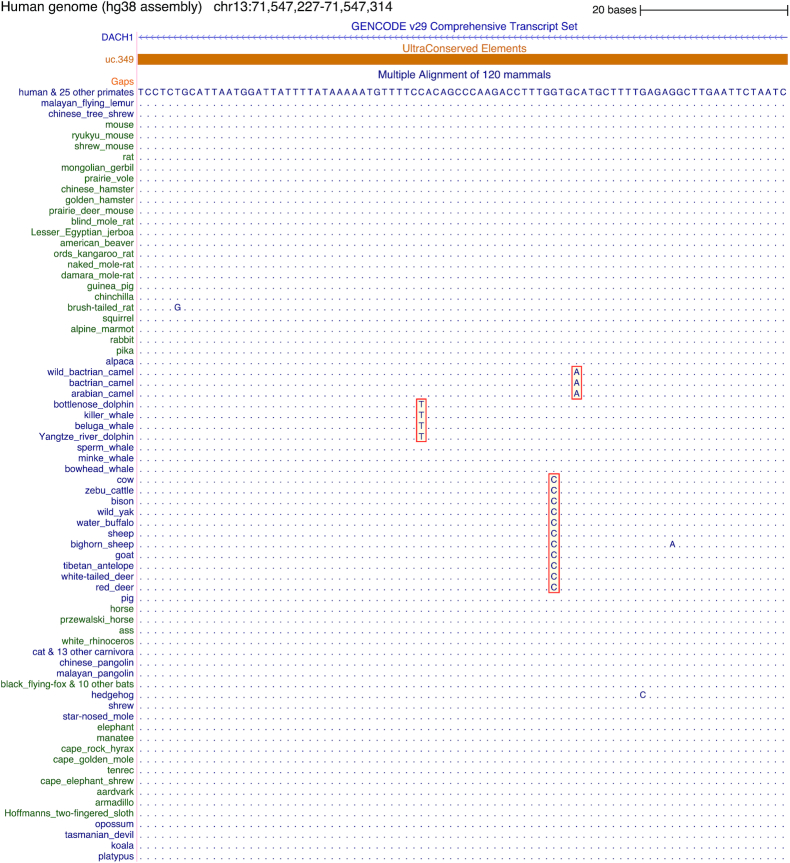
Example of a sequence alignment of 120 mammals showing an 88-bp region inside a UCE. This UCE is located in an intron of the *DACH1* gene, which encodes a transcription factor important for development. Dots in the 120-mammal alignment refer to bases that are identical to those in the human genome. For space reasons, 25 primates, 13 carnivora, and 10 bats that all have identical sequence to human are not shown. Green and blue fonts indicate species of different clades. The alignment of this ultraconserved region shows that most columns are identical across all 120 mammals but also reveals a few substitutions. Some of these substitutions are species-specific and may be attributed to base errors in the assembly. Other substitutions are shared among independently sequenced genomes of related species (red boxes), which makes base errors very unlikely. We used shared substitutions to calculate a lower bound for the percentage of UCE positions that can vary across placental mammals. We used both shared and species-specific substitutions to calculate an upper bound for this percentage.

Here, we sought to quantify the variability of UCEs among placental mammals. However, accurately estimating sequence variability in these highly conserved regions is not straightforward because base errors in genome assemblies can mimic real mutations [32, 34, 41, 42]. Such base errors would overestimate the true variability within UCEs. To address this problem, we utilized the increased taxonomic sampling in our alignment to compute an upper and a lower bound of the number of alignment columns that exhibit a substitution. To compute a lower bound, we considered an alignment column as variable only if the same substitution is shared among ≥2 related sister species (Fig. 2). Because the genomes of 2 related sister species were independently sequenced and assembled, the presence of a shared substitution makes a base error in the assembly very unlikely. To compute an upper bound, we considered a column as variable if ≥1 substitution occurred (Fig. 2), regardless of whether this substitution is shared among related species or is species-specific. For robustness, we limited our analysis to the 441 of 480 UCEs for which we aligned ≥110 placental mammals.

**Figure 3:**
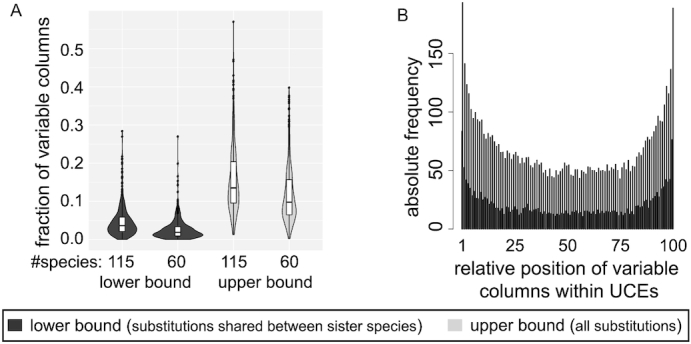
Variability of UCEs across placental mammals. For each alignment position in the 441 UCEs for which ≥110 placental mammals had aligning sequence in our genome alignment, we examined whether positions in the UCE are identical or were substituted at least once across the 115 non-human placental mammals. (A) Violin and box plots show the distribution of the fraction of variable positions per UCE across placental mammals. The white box spans the first to the third quartile, the middle line indicates the median. In addition to considering all 115 non-human placental mammals, we also determined the fraction of variable positions per UCE considering only 60 non-human placental mammals. This illustrates that analyzing fewer species would underestimate UCE variability. (B) Bar plots show the number of substitutions observed in UCEs with respect to their relative position in UCEs. UCEs were divided into 100 equally sized bins. Both upper and lower bounds show that UCEs are more variable at their flanks than in their center.

Considering all nucleotide changes (upper bound), we found that on average 15.6% (median 13.5%) of the columns of a UCE contain ≥1 nucleotide change (Fig. 3A, Supplementary Table 2). Using the more robust lower bound for nucleotide changes, we found that on average 4.7% (median 3.6%) of the UCE columns are variable. None of the UCEs is perfectly conserved across placental mammals based on the upper bound, which considers all nucleotide changes. Considering only 60 instead of all 115 non-human placental mammals in this analysis, we obtained average upper and lower bound estimates of 11.8% (median 9.8%) and 2.7% (median 1.9%), respectively (Fig. 3A), indicating that analyzing fewer species would underestimate UCE variability. Our 120-mammal analysis shows that UCEs contain on average between 4.7% and 15.6% variable alignment columns across placental mammals and provides the first quantification of evolutionary variability within UCEs.

To investigate factors associated with UCE variability, we first tested whether there is a correlation between the percentage of variable columns and the length of UCEs. We found a weak but significant negative correlation (Kendall τ of −0.11 and −0.12 for the lower and upper bound variability, both *P*-values < 10^−3^; Supplementary Fig. 2), indicating that longer UCEs tend to have a lower percentage of variable columns. We further assessed whether positions exhibiting substitutions are uniformly distributed within UCEs. To account for the variable length of UCEs, we divided each UCE into 100 equally sized bins and computed the cumulative number of UCEs with substitutions per relative position. Interestingly, using our lower and upper bound estimation, we consistently found that the center regions of UCEs exhibit the fewest variable alignment columns (Fig. 3B), suggesting that the center region is most constrained.

### Case study 2: Evolution of placental mammal-specific enhancers

An increasing body of evidence suggests that changes in gene regulatory elements such as enhancers are important for phenotypic evolution [28, 30, 50–53]. The evolutionary origin of enhancers can sometimes be linked to the origin of lineage-specific traits. For example, gain of enhancers in mammals has been linked to the emergence of the neocortex [54], enhancer gain near neurogenesis-regulating genes in humans has been linked to the expansion of the human neocortex [55], and gains of enhancers near hair-related genes in mammals coincide with the origin of body hair [56]. Here, we used our 120-mammal alignment to identify enhancers whose sequence is only conserved among placental mammals. To assess the conservation of enhancers, we screened FANTOM enhancers [57] for conserved 10-mers, which roughly reflects the size of a transcription factor binding site motif [58].

As a proof of principle, we first identified 1,820 FANTOM enhancers with ≥1 10-mer that is conserved across all mammalian families including marsupials and the monotreme platypus. Using GREAT [59], we found that these enhancers are significantly associated with genes involved in a variety of developmental processes (Supplementary Tables 3 and 4). This is consistent with previous findings that enhancers, which arose in the mammalian ancestor or earlier, are associated with developmental genes [56].

To identify placental mammal–specific enhancers, we determined which FANTOM enhancers have ≥1 conserved 10-mer in all major placental mammal clades but have no aligning sequence in marsupials and the platypus. Based on this definition, 658 FANTOM enhancers are conserved and emerged in placental mammals (Supplementary Table 5). Interestingly, we found that these enhancers exhibit, among other categories, significant association with placenta-related genes. For example, the top-enriched Mouse Genome Informatics (MGI) Mouse Phenotype term is “abnormal placenta labyrinth morphology” (MP:0001716) and Gene Ontology (GO) biological process terms “embryonic placenta development” (GO:0001892) and “labyrinthine layer blood vessel development” (GO:0060716) are significantly enriched (Supplementary Tables 6 and 7). Consistently, 149 of 658 (23%) of these placental mammal–specific enhancers overlap predicted placenta enhancers [60].

Next, we investigated whether the set of conserved 10-mer sequences of the 1,820 mammal-conserved and 658 placental mammal–specific enhancers are enriched in transcription factor–binding motifs. Using Analysis of Motif Enrichment (AME) from the MEME suite [61, 62], we found enrichments for motifs of several ETS (E26 transformation-specific) and AP-1 (activating protein-1) transcription factors in both 10-mer sets (Supplementary Tables 8 and 9). These transcription factors play various roles in development, cell proliferation, and differentiation [63, 64]. In agreement with GO “artery morphogenesis” (GO:0048844) and MGI “abnormal artery development” (MP:0003410) gene enrichments of mammal-conserved enhancers (Supplementary Tables 3 and 4) and the GO “labyrinthine layer blood vessel development” (GO:0060716) gene enrichment of placental mammal–specific enhancers (Supplementary Table 7), ETS family members FLI1, ERG, and ETV2, whose motifs are enriched in the 10-mer sets, are involved in hematopoiesis and endothelial development [65, 66]. Interestingly, AP-1 family members JUN, JunB, and FOS, whose motifs are also enriched in the 10-mer sets, are involved in trophoblast cell invasion into the uterus and essential for placentation [67, 68]. This agrees with placenta-related gene enrichments of placental mammal–specific enhancers (Supplementary Tables 6 and 7) and supports placenta-related functions of these enhancers. Furthermore, 10-mers in the placental mammal–specific enhancers exhibit enriched motifs for FOXP3 (forkhead-box-protein P3). This transcription factor has been linked to pre-eclampsia, a pregnancy-related disorder characterized by high blood pressure [69, 70].

Together, our analysis suggests that a subset of enhancers that emerged in placental mammals may have been involved in the evolution of placental mammal–specific aspects of the placenta. These enhancers could serve as a starting point for more elaborate studies on the molecular basis of placenta evolution.

### Summary

We generated a multiple-genome alignment comprising 120 mammals and used this alignment to project human genes to 119 other mammalian genomes. To exemplify how our alignment may facilitate comparative genomics studies, we quantified the variability within ultraconserved elements and showed that placental mammal–specific enhancers are significantly associated with placenta-related genes. The multiple-genome alignment, sets of conserved elements, and comparative gene annotations are a valuable resource for further studies, which can be visualized in a UCSC genome browser installation [38].

## Materials and Methods

### Phylogeny

The order level of the phylogeny is based on dos Reis et al. [71]. The primate phylogeny is based on Perelman et al. [72]. Rodents were placed on the basis of Fabre et al. [73]. We based the Afrotheria phylogeny on Meredith et al., Poulakakis et al., and O'Leary et al. [74–76]. Sorex, Erinaceus, and Condylura were placed on the basis of Brace et al. [77]. The Carnivora phylogeny is based on Meredith et al. and Flynn et al. [74, 78]. Artiodactyla is based on O'Leary et al. and Ropiquet et al. [76, 79]. The Chiroptera phylogeny is based on Teeling et al. and Agnarsson et al. [80, 81].

### Genome alignment

To compute pairwise and multiple genome alignments, we used the human hg38 assembly as the reference (Supplementary Fig. 1 shows the entire workflow). We first built pairwise alignments between human and a query species using lastz and axtChain to compute co-linear alignment chains [82]. To align placental mammals, we used previously determined lastz parameters (K = 2400, L = 3000, Y = 9400, H = 2000, and the lastz default scoring matrix) that have a sufficient sensitivity to capture orthologous exons [16]. To align chimpanzee, bonobo, and gorilla, we changed the lastz parameters (K = 4500 and L = 4500).

After building chains, we applied RepeatFiller (RRID:SCR_017414), a method that performs another round of local alignment, considering unaligning regions ≤20 kb in size that are bounded by co-linear alignment blocks up- and downstream. RepeatFiller removes any repeat masking from the unaligned region and is therefore able to detect novel alignments between repetitive regions. We have previously shown that RepeatFiller detects several megabases of aligning repetitive sequences that would be missed otherwise. After RepeatFiller, we applied chainCleaner with parameters -LRfoldThreshold = 2.5 -doPairs -LRfoldThresholdPairs = 10 -maxPairDistance = 10000 -maxSuspectScore = 100000 -minBrokenChainScore = 75000 to improve alignment specificity. Pairwise alignment chains were converted into alignment nets using a modified version of chainNet that computes real scores of partial nets. Nets were filtered using NetFilterNonNested.perl with parameters -doUCSCSynFilter -keepSynNetsWithScore 5000 -keepInvNetsWithScore 5000, which applies the UCSC “syntenic net” score thresholds (minTopScore of 300000 and minSynScore of 200000) and keeps nested nets that align to the same locus (inversions or local translocations; net type “inv” or “syn” according to netClass) if they score ≥5,000. For the Mongolian gerbil, tarsier, Malayan flying lemur, sperm whale, Przewalski's horse, Weddell seal, Malayan pangolin, Chinese pangolin, Hoffmann's two-fingered sloth, and Cape rock hyrax that have genome assemblies with a scaffold N50 ≤1,000,000 and a contig N50 ≤100,000, we just required that nets have a score ≥100,000. For marsupials and platypus, we lowered the score threshold for nets to 10,000 and kept inv or syn nets with scores ≥3,000. Next, we used the filtered nets to compute a human-referenced multiple genome alignment with MULTIZ-tba. Finally, to distinguish between unaligning genomic regions that are truly diverged and genomic regions that do not align because they overlap assembly gaps in the query genome [83], we post-processed the multiple-genome alignment and removed all unaligning regions (e-lines in a maf block) that either overlap an assembly gap in the respective query genome(s) or are not covered by any alignment chain.

The main difference between this 120-mammal alignment and our previous 144-vertebrate alignment [16] is that the former focuses entirely on mammals and includes many new species (120 vs 74 mammals, see Supplementary Table 1). In addition, we updated genome assemblies of 12 species that were already included in the previous alignment (species are marked in Supplementary Table 1). Finally, the 120-mammal alignment used RepeatFiller to improve the completeness of alignments between repetitive regions.

### Identification of conserved regions

We used msa_view to extract 4-fold degenerated codon positions based on the human RefSeq gene annotation and used PhyloFit [84] to estimate the length of all branches in the tree as substitutions per neutral site. This tree was used to detect constrained elements with PhastCons [18] and GERP++ (GERP, RRID:SCR_000563) [43]. For running PhastCons, we used the parameters rho = 0.31, expected-length = 45, and target-coverage = 0.3. For GERP++, we used default parameters.

### Comparative gene annotation with CESAR

Genes were annotated using the CESAR gene annotation pipeline [15, 39, 40] using all protein-coding transcripts from the human ENSEMBL 96 gene annotation as input [85]. To count the number of annotated genes per species, we first extracted per locus the transcript with the longest open reading frame (ignoring all shorter overlapping transcripts) and then determined the number of unique gene symbols.

### UCE divergence analysis

UCE coordinates were downloaded from UCbase2.0 [86]. We converted the coordinates of the 481 UCEs from hg19 to hg38 using liftOver. We merged UCE 208 and 209 into 1 UCE because they are directly adjacent. We then extracted alignments of UCEs from our 120-mammal alignment. For robustness, we only considered the 441 UCEs for which we aligned ≥110 of placental mammals over the entire length of the UCE and further removed sequences that contained assembly gaps. Next, we used a previously developed bottom-up Fitch-like parsimony approach [87] to identify alignment columns containing ≥1 substitution. To account for the possibility of base errors in assemblies, we additionally identified alignment columns that have shared substitutions between ≥2 sister species. We used shared substitutions as a lower bound estimate for variable columns in UCE alignments. To investigate the influence of the number of considered species, we repeated this analysis for the same 441 UCEs but considered only 60 instead of all 115 non-human placental mammals (marked in Supplementary Table 1).

To investigate how variable positions are distributed within UCEs, we had to account for the different lengths of UCEs. To this end, we normalized the positions of each UCE into 100 equally sized bins. Because not all positions can be uniquely assigned to a single bin (unless the UCE length is a multiple of 100), we duplicated the value for each position in a UCE (1 for nucleotide change, 0 otherwise) 100 times and then grouped them into bins. The cumulative value of each bin was then normalized by bin size (length of the UCE) to obtain a per-UCE value for nucleotide changes at each relative position.

### Analysis of FANTOM enhancers

We downloaded the coordinates of the 38,548 robust FANTOM enhancers from SlideBase [57, 88]. Coordinates were then mapped from the human hg19 genome assembly to hg38 using liftOver. Next, we identified the most conserved 10-mers in all FANTOM enhancers using a sliding-window approach. We then counted the number of species that were aligned with identical 10-mers per following clades: Primatomorpha, Glires, Artiodactyla, Ferae, Chiroptera, Eulipotyphla, Atlantogenata, and non-placental mammals. We defined an enhancer as conserved across all mammals if ≥50% of the species in each of these clades were aligned with an identical 10-mer. For identifying placental mammal–specific enhancers, we required that ≥50% of the species in each placental mammal clade be aligned with an identical 10-mer and that no sequence be aligned to the entire enhancer region for any non-placental mammal.

### Enrichment analysis for placental mammal–specific enhancers

We used the GREAT webserver version 4 (19 August 2019) to test whether placental mammal–specific enhancers are enriched near genes belonging to certain functional groups [59]. We used the hg38 genome assembly coordinates and the 38,548 robust FANTOM enhancers as background [57]. We considered terms significantly enriched if they exceed a 2-fold enrichment (RegionFoldEnrich), are associated with ≥5 genes, and exhibit a corrected *P*-value (hypergeometric false discovery rate Q-value) <0.05. In addition to the enrichment analysis, we downloaded predicted placenta enhancers from reference [60] and compared how many placental mammal–specific enhancers overlap predicted placenta enhancers. Here, we required that ≥50% of the enhancer overlaps a predicted placenta enhancer.

### Motif enrichment analysis of conserved 10-mers

To identify enriched transcription factor–binding motifs for mammal-conserved and placental mammal–specific enhancers, we first identified all conserved 10-mers in each enhancer set using the same criteria as described above and merged overlapping 10-mers. The human sequences of these merged 10-mers were then used as input for AME from the MEME suite (RRID:SCR_001783) [61, 62]. Shuffled sequences were used as background, and motifs with an e-value <0.05 were considered as enriched.

## Availability of Supporting Data and Materials

The 120-mammal alignment, phylogenetic tree, conserved elements, GERP and PhastCons tracks, and CESAR gene annotations for 119 non-human mammals are available for download [89]. These data can also be loaded as a trackhub [90] into the UCSC genome browser via [91]. Furthermore, our UCSC genome browser installation [38] visualizes all data. Snapshots of the data and code are also archived in the *GigaScience* GigaDB repository [92].

## Additional Files

**Supplementary Figure 1:** Genome alignment workflow. Input genome assemblies are indicated by light blue rectangles, intermediate data (chains and nets) by gray rectangles, and the resulting multiple sequence alignment by a golden rectangle. Red ellipses depict the tools that were used for computing the alignments: lastz is used for computing local pairwise alignments between the human genome assembly and each of the 119 other mammal genome assemblies; axtChain [1] extracts co-linear local alignments that occur in the same order and same strand on a reference and a query chromosome and builds pairwise co-linear alignment chains; RepeatFiller [2] and chainCleaner [3] improve the pairwise alignment chains; chainNet [1] generates pairwise alignment nets by building a hierarchical collection of chains or parts of chains such that each locus in the reference is covered by ≥1 alignment to the query; and NetFilterNonNested [3] removes low-scoring and non-syntenic parts of nets in a non-nested fashion to generate the final filtered pairwise alignments. These 119 pairwise alignments are the input for MultiZ [4], which computes the multiple sequence alignment of 120 mammals.

**Supplementary Figure 2:** Relationship between the variability and length of UCEs. Scatter plots show that there is a weak negative correlation between the fraction of variable columns and the length of UCEs. (A) For the lower bound value for the fraction of variable columns (only considering shared substitutions), we obtain Kendall τ of –0.11 with *P*-value < 10^–3^. (B) For the upper bound value for the fraction of variable columns (considering all substitutions), we obtain Kendall τ of –0.12 with *P*-value < 10^–3^. This indicates that larger UCEs tend to be slightly less variable than smaller UCEs. Kendall τ is preferred over Spearman rank correlation if the data contain ties.

**Supplementary Table 1:** Species and genome assemblies used in the alignment. The previous 145-vertebrate alignment in column G refers to Sharma and Hiller (2017) [16].

**Supplementary Table 2:** Fraction of variable alignment columns per UCE. Coordinates refers to the human hg38 genome assembly.

**Supplementary Table 3:** GREAT enrichments of enhancers conserved across mammals for mouse phenotypes.

**Supplementary Table 4:** GREAT enrichments of enhancers conserved across mammals for Gene Ontology biological processes.

**Supplementary Table 5:** FANTOM enhancers that are placental mammal specific and contain ≥1 conserved 10-mer.

**Supplementary Table 6:** GREAT enrichments of placental mammal enhancers for mouse phenotypes. Placenta-related terms are in boldface.

**Supplementary Table 7:** GREAT enrichments of placental mammal enhancers for Gene Ontology biological processes. Placenta-related terms are in boldface.

**Supplementary Table 8:** Enriched motifs in 10-mers of placental mammal–specific enhancers.

**Supplementary Table 9:** Enriched motifs in 10-mers of mammal conserved enhancers.

giz159_GIGA-D-19-00313_Original_Submission

giz159_GIGA-D-19-00313_Revision_1

giz159_Response_to_Reviewer_Comments_Original_Submission

giz159_Reviewer_1_Report_Original_Submission Hsin-Nan Lin -- 10/30/2019 Reviewed

giz159_Reviewer_1_Report_Revision_1 Hsin-Nan Lin -- 12/2/2019 Reviewed

giz159_Reviewer_2_Report_Original_Submission Quan Nguyen -- 11/6/2019 Reviewed

giz159_Supplemental_Files

## Abbreviations

AME: Analysis of Motif Enrichment; bp: base pairs; CESAR: Coding Exon-Structure Aware Realigner; FANTOM: Functional Annotation of the Mammalian Genome; GO: Gene Ontology; GREAT: Genomic Regions Enrichment of Annotations Tool; MGI: Mouse Genome Informatics; UCE: ultraconserved element; UCSC: University of California Santa Cruz.

## Competing Interests

The authors declare that they have no competing interests.

## Funding

This work was supported by the Max Planck Society and the Leibniz Association (SAW-2016-SGN-2).

## Authors' Contributions

MH and NH conceived the study. NH generated and analyzed all data. MH and NH wrote the manuscript and produced the figures.
