# Supplementary material for: A genome alignment of 120 mammals highlights ultraconserved element variability and placenta-associated enhancers
Source: Gigascience. 2020 Jan 3;9(1):giz159. doi: 10.1093/gigascience/giz159 (PMC6941714; doi:10.1093/gigascience/giz159)
Supplement: giz159_GIGA-D-19-00313_Revision_1 [file giz159_giga-d-19-00313_revision_1.pdf]

GigaScience

A genome alignment of 120 mammals highlights ultraconserved element variability and placenta associated enhancers

--Manuscript Draft--

**Manuscript Number:**	GIGA-D-19-00313R1
**Full Title:**	A genome alignment of 120 mammals highlights ultraconserved element variability and placenta associated enhancers
**Article Type:**	Data Note
**Funding Information:**	Max-Planck-Gesellschaft (-) — Dr. Michael Hiller<br>Leibniz-Gemeinschaft (SAW-2016-SGN-2) — Dr. Michael Hiller
**Abstract:**	Multiple alignments of mammalian genomes have been the basis of many comparative genomic studies aiming at annotating genes, detecting regions under evolutionary constraint, and studying genome evolution. A key factor that affects the power of comparative analyses is the number of species included in a genome alignment. To utilize the increased number of sequenced genomes and to provide an accessible resource for genomic studies, we generated a mammalian genome alignment comprising 120 species. We used this alignment and the CESAR method to provide protein-coding gene annotations for 119 non-human mammals. Furthermore, we illustrate the utility of this alignment by two exemplary analyses. First, we quantified how variable ultraconserved elements (UCEs) are among placental mammals. Leveraging the high taxonomic coverage in our alignment, we estimate that UCEs contain on average between 4.7% and 15.6% variable alignment columns. Furthermore, we show that the center regions of UCEs are generally most constrained. Second, we identified enhancer sequences that are only conserved in placental mammals. We found that these enhancers are significantly associated with placenta-related genes, suggesting that some of these enhancers may be involved in the evolution of placental mammal-specific aspects of the placenta. The 120-mammal alignment and all other data are available for analysis and visualization in a genome browser at https://genome-public.pks.mpg.de/ and for download at https://bds.mpi-cbg.de/hillerlab/120MammalAlignment/ .
**Corresponding Author:**	Michael Hiller<br><br>GERMANY
**Corresponding Author Secondary Information:**	
**Corresponding Author's Institution:**	
**Corresponding Author's Secondary Institution:**	
**First Author:**	Nikolai Hecker
**First Author Secondary Information:**	
**Order of Authors:**	Nikolai Hecker<br>Michael Hiller
**Order of Authors Secondary Information:**	
**Response to Reviewers:**	Nov 29th, 2019<br>Dear Dr. Edmunds,<br><br>Thank you very much for considering a revised version of our manuscript “A genome alignment of 120 mammals highlights ultraconserved element variability and placenta associated enhancers”.<br><br>We would like to thank all both reviewers for their insightful comments. We have now

Powered by Editorial Manager® and ProduXion Manager® from Aries Systems Corporation

	addressed all points raised and revised the manuscript accordingly. In particular, we have<br>•performed an analysis of enriched transcription factor binding motifs, which revealed enrichments of factors that are likely relevant for vasculature and placenta-related gene enrichments associated with the respective enhancer sets (new Supplementary Tables 8 and 9),<br>•analyzed a genome alignment where the number of species was subsampled to 50%, which showed that a reduced number of species would underestimate UCE variability (updated Figure 3A),<br>•show the UCE length is negatively correlated with variability (new Supplementary Figure 2),<br>•made several text changes to clarify the definitions of conserved elements and ultraconserved elements, and provide more background for the methods used to detect conserved genomic regions,<br>•added a workflow of how the multiple genome alignment was generated (new Supplementary Figure 1),<br>•and added alignments of the UCE sequences in fasta format.<br><br>To make it really easy to use and browse our whole genome alignment of 120 mammals and the gene annotations of 119 non-human species, we have now added all data to a public UCSC genome browser installation that is freely accessible at https://genome-public.pks.mpg.de/.<br><br><br>We would kindly like to ask that the UCE alignments in FASTA format (https://bds.mpi-cbg.de/hillerlab/120MammalAlignment/Human120way/data/uce/) will be incorporated into the GigaDB entry associated with our manuscript.<br><br>Text changes are highlighted in red font in the manuscript. Our point-by-point response to the comments raised by the reviewers is uploaded as a separate Word document (labeled as Supplement, since there is no other appropriate category).<br><br>We have no new software to register.<br><br>We hope that our revised manuscript is now acceptable for publication. We look forward to hearing from you.<br><br>Sincerely,<br>Michael Hiller
**Additional Information:**	
**Question**	**Response**
Are you submitting this manuscript to a special series or article collection?	No
**Experimental design and statistics**<br><br><br>Full details of the experimental design and statistical methods used should be given in the Methods section, as detailed in our Minimum Standards Reporting Checklist. Information essential to interpreting the data presented should be made available in the figure legends.<br><br><br>Have you included all the information requested in your manuscript?	Yes

Powered by Editorial Manager® and ProduXion Manager® from Aries Systems Corporation

**Resources**<br><br>A description of all resources used, including antibodies, cell lines, animals and software tools, with enough information to allow them to be uniquely identified, should be included in the Methods section. Authors are strongly encouraged to cite Research Resource Identifiers (RRIDs) for antibodies, model organisms and tools, where possible.<br><br>Have you included the information requested as detailed in our Minimum Standards Reporting Checklist?	Yes
**Availability of data and materials**<br><br>All datasets and code on which the conclusions of the paper rely must be either included in your submission or deposited in publicly available repositories (where available and ethically appropriate), referencing such data using a unique identifier in the references and in the "Availability of Data and Materials" section of your manuscript.<br><br>Have you have met the above requirement as detailed in our Minimum Standards Reporting Checklist?	Yes

Powered by Editorial Manager® and ProduXion Manager® from Aries Systems Corporation

Manuscript
Click here to access/download;Manuscript;Manuscript_changesTracked.docx
Click here to view linked References

A genome alignment of 120 mammals highlights ultraconserved element variability and placenta associated enhancers

Nikolai Hecker [1,2,3] and Michael Hiller [1,2,3*]

[1]Max Planck Institute of Molecular Cell Biology and Genetics, Dresden, Germany
[2]Max Planck Institute for the Physics of Complex Systems, Dresden, Germany
[3]Center for Systems Biology Dresden, Germany

*To whom correspondence should be addressed:
Michael Hiller
Computational Biology and Evolutionary Genomics, Max Planck Institute of Molecular Cell Biology and Genetics & Max Planck Institute for the Physics of Complex Systems, Dresden, Germany.
Tel: +49 351 210 2781
Fax: +49 351 210 1209
Email: hiller@mpi-cbg.de

ORCID IDs:
Nikolai Hecker: 0000-0003-1693-4257
Michael Hiller: 0000-0003-3024-1449

Running title: Whole-genome alignment of 120 mammals

Keywords: genome alignment, comparative gene annotation, ultraconserved elements, enhancers, mammals

Abstract

Multiple alignments of mammalian genomes have been the basis of many comparative genomic studies aiming at annotating genes, detecting regions under evolutionary constraint, and studying genome evolution. A key factor that affects the power of comparative analyses is the number of species included in a genome alignment. To utilize the increased number of sequenced genomes and to provide an accessible resource for genomic studies, we generated a mammalian genome alignment comprising 120 species. We used this alignment and the CESAR method to provide protein-coding gene annotations for 119 non-human mammals. Furthermore, we illustrate the utility of this alignment by two exemplary analyses. First, we quantified how variable ultraconserved elements (UCEs) are among placental mammals. Leveraging the high taxonomic coverage in our alignment, we estimate that UCEs contain on average between 4.7% and 15.6% variable alignment columns. Furthermore, we show that the center regions of UCEs are generally most constrained. Second, we identified enhancer sequences that are only conserved in placental mammals. We found that these enhancers are significantly associated with placenta-related genes, suggesting that some of these enhancers may be involved in the evolution of placental mammal-specific aspects of the placenta. The 120-mammal alignment and all other data are available for analysis and visualization in a genome browser at https://genome-public.pks.mpg.de/ and for download at https://bds.mpi-cbg.de/hillerlab/120MammalAlignment/.

Introduction

Comparative genomics has substantially contributed to detecting and classifying functional regions in genomes and understanding genome evolution [1, 2]. A foundation for most comparative genomics analyses are alignments between entire genomes. Several computational methods rely on genome alignments for annotating coding and non-coding genes, and genome alignments have been used to detect novel coding exons, revise exon-intron boundaries and correct the positions of annotated start or stop codons [3-9]. Many gene or exon finders utilize genome alignments to increase the reliability of their predictions [10-14]. In addition, genome alignments provide an effective way to project genes from a reference species annotation to aligned (query) species [15-17]. Genome alignments have also been used to identify regions that evolve under purifying selection and thus likely have a biological function [18, 19]. Around 3-15% of the human genome is estimated to be evolutionarily constrained [20], and most of the constraint detected in genome alignments is located in conserved non-exonic elements that often overlap *cis*-regulatory elements such as enhancers [21, 22]. Furthermore, genome alignments have been instrumental for understanding the evolution of genomes, which uncovered genomic determinants of trait differences [23-30], and provided insights into evolutionary history and species' biology [31-34].

A key factor affecting the power of comparative analyses is the number of species included in the genome alignment. Since higher taxonomic coverage increases the power to detect evolutionary constraint [35] and yields more robust results in phylogenetic and evolutionary studies [36, 37], it is desirable to include many sequenced genomes to capture the diversity of species in a respective clade. While the availability of sequenced genomes was a limiting factor in the past, advances in sequencing and assembly technology have led to a wealth of sequenced genomes, illustrated by the availability of more than 100 mammalian genomes.

To provide a comparative genomics resource that reflects the increased availability of sequenced mammals and is easily accessible to genomic experts and non-experts, we generated a multiple genome alignment of 120 mammals. We used the human gene annotation and Coding Exon-Structure Aware Realigner (CESAR) to provide comparative gene annotations for all 119 non-human mammals. Furthermore, we demonstrate the utility of the high species coverage in our alignment by (i) quantifying how variable ultraconserved elements are among placental mammals and (ii) identifying *cis*-regulatory elements (enhancers) that arose in the placental mammal lineage and showing that these enhancers are significantly associated with placenta-related genes. To facilitate

comparative analyses using our resources, we provide the multiple genome alignment, a phylogenetic tree, conserved regions including GERP++ and PhastCons conservation scores, and the comparative gene annotations in a UCSC genome browser installation at https://genome-public.pks.mpg.de/

Results and Discussion

Generating a multiple genome alignment of 120 mammals

To compute a comprehensive multiple genome alignment of mammals, we used human as the reference species and aligned 119 non-human mammals that have genome assemblies with a scaffold N50 value of at least 100,000 (Supplementary Table 1). The phylogeny of these 120 species is shown in Figure 1. The workflow and methods used to compute the alignment are shown in Supplementary Figure 1.

Comparative gene and conserved element annotation

We first used our alignment to annotate protein-coding genes in all 119 non-human mammals. To this end, we used CESAR [15, 38, 39] to project all coding exons of human genes and annotated intact exons in all 119 non-human aligned mammals. Intact exons are defined as having an intact open reading frame without premature stop codons, and two consensus splice sites (internal exons) or one consensus splice site and a start (first exon) or stop (last codon) codon. Since intact exons can be missing due to assembly gaps and assembly base errors [32, 34, 40, 41], we determined for each species the number of genes where at least one intact exon was annotated. We found that between 15,868 and 18,047 of the human genes have at least one intact exon alignment in placental mammals (Figure 1). For marsupials, we annotated between 15,119 and 16,259 genes. In the platypus, a member of the monotremes, we annotated 9,669 genes (Figure 1).

Second, in addition to annotating protein-coding genes, we annotated genomic regions that likely evolve under evolutionary constraint (purifying selection). To this end, we used PhastCons [18], a phylogenetic Hidden Markov Model method, and GERP++ [42], a method that directly measures the number of substitutions per site that were rejected by purifying selection. We applied both methods to detect regions constrained across all mammals in our alignment. PhastCons and GERP++ identified 13,257,408 and 1,612,714 conserved elements covering 5.5% and 9.9% of the human genome, respectively.

Case study 1: Quantifying divergence in ultraconserved elements

The large number of mammalian species in our genome alignment provides an opportunity to quantify how variable highly conserved genomic elements are across placental mammals. We focused on a subset of highly conserved elements, called ultraconserved elements (UCEs), that have attracted much attention as deletions of several of these elements does not affect cellular fitness and resulted in viable organisms [43-45]. UCEs were originally defined as genomic regions that are at least 200 bp long (the largest UCE is 779 bp long) and have identical sequences between human, mouse and rat [46]. Despite the fact that only three mammals were used to identify these genomic regions, UCEs are also highly conserved in other mammals and typically align to non-mammalian vertebrates [47]. For example, human UCE sequences align to chicken with an average sequence identity of 96% [46]. Transgenic enhancer assays have shown that many non-exonic UCEs overlap regulatory elements that drive gene expression during development [22] and a recent study showed that ultraconserved enhancers are required for normal development in mice [44]. UCEs are not mutational cold spots as there is genetic variation in the human population; however, derived mutations are under strong purifying selection [48].

Here, we sought to quantify the variability of UCEs among placental mammals. However, accurately estimating sequence variability in these highly-conserved regions is not straightforward as base errors in genome assemblies can mimic real mutations [32, 34, 40, 41]. Such base errors would overestimate the true variability within UCEs. To address this problem, we utilized the increased taxonomic sampling in our alignment to compute an upper and a lower bound of the number of alignment columns that exhibit a substitution. To compute a lower bound, we only considered an alignment column as variable if the same substitution is shared among at least two related sister species (Figure 2). Since genomes of two related sister species were independently sequenced and assembled, the presence of a shared substitution makes a base error in the assembly very unlikely. To compute an upper bound, we considered a column as variable if at least one substitution occurred (Figure 2), regardless of whether this substitution is shared among related species or species-specific. For robustness, we limited our analysis to the 441 of 480 UCEs for which we aligned at least 110 placental mammals.

Considering all nucleotide changes (upper bound), we found that on average 15.6% (median 13.5%) of the columns of a UCE contain at least one nucleotide change (Figure 3A, Supplementary Table 2). Using the more robust lower bound for nucleotide changes, we found that on average 4.7% (median 3.6%) of the UCE columns are variable. None of the UCEs is perfectly conserved across placental mammals based on the upper bound

which considers all nucleotide changes. Considering only 60 instead of all 115 non-human placental mammals in this analysis, we obtained average upper and lower bound estimates of 11.8% (median 9.8%) and 2.7% (median 1.9%), respectively (Figure 3A), indicating that fewer species underestimate UCE variability. Our 120-mammal analysis shows that UCEs contain on average between 4.7% and 15.6% variable alignment columns across placental mammals and provides the first quantification of evolutionary variability within UCEs.

To investigate factors associated with UCE variability, we first tested whether there is a correlation between the percentage of variable columns and the length of UCEs. We found a weak but significant negative correlation (Kendall's tau of -0.11 and -0.12 for the lower and upper bound variability, both p-values < 10^{-3}; Supplementary Figure 2), indicating that longer UCEs tend to have a lower percentage of variable columns. We further assessed whether positions exhibiting substitutions are uniformly distributed within UCEs. To account for the variable length of UCEs, we divided each UCE into 100 equally sized bins and computed the cumulative number of UCEs with substitutions per relative position. Interestingly, using our lower and upper bound estimation, we consistently found that the center region of UCEs exhibit the lowest number of variable alignment columns (Figure 3B), suggesting that the center region is most constrained.

Case study 2: Evolution of placental mammal-specific enhancers

An increasing body of evidence suggests that changes in gene regulatory elements such as enhancers are important for phenotypic evolution [28, 30, 49-52]. The evolutionary origin of enhancers can sometimes be linked to the origin of lineage-specific traits. For example, gain of enhancers in mammals has been linked to the emergence of the neocortex [53], enhancer gain near neurogenesis-regulating genes in humans has been linked to the expansion of the human neocortex [54], and gains of enhancers near hair-related genes in mammals coincides with the origin of body hair [55]. Here, we used our 120 mammal alignment to identify enhancers whose sequence is only conserved among placental mammals. To assess the conservation of enhancers, we screened FANTOM enhancers [56] for conserved 10-mers, which roughly reflects the size of a transcription factor binding site motif [57].

As a proof of principle, we first identified 1,820 FANTOM enhancers with at least one 10-mer that is conserved across all mammalian families including marsupials and the monotreme platypus. Using GREAT [58], we found that these enhancers are significantly associated with genes involved in a variety of developmental processes (Supplementary Tables 3 and 4). This is consistent with previous findings that enhancers, which arose in mammalian ancestor or earlier, are associated with developmental genes [55].

To identify placental mammal specific enhancers, we determined which FANTOM enhancers have at least one conserved 10-mer in all major placental mammal clades but have no aligning sequence in marsupials and the platypus. Based on this definition, 658 FANTOM enhancers are conserved and emerged in placental mammals (Supplementary Table 5). Interestingly, we found that these enhancers exhibit, among other categories, significant association with placenta-related genes. For example, the top-enriched MGI Mouse Phenotype term is 'abnormal placenta labyrinth morphology' (MP:0001716) and Gene Ontology (GO) biological process terms 'embryonic placenta development' (GO:0001892) and 'labyrinthine layer blood vessel development' (GO:0060716) are significantly enriched (Supplementary Tables 6 and 7). Consistently, 149 of 658 (23%) of these placental mammal-specific enhancers overlap predicted placenta enhancers [59].

Next, we investigated whether the set of conserved 10-mer sequences of the 1,820 mammal-conserved and 658 placental mammal-specific enhancers are enriched in transcription factor binding motifs. Using Analysis of Motif Enrichment (AME) from the MEME suite [60, 61], we found enrichments for motifs of several ETS (E26 transformation-specific) and AP-1 (activating protein-1) transcription factors in both 10-mer sets (Supplementary Tables 8 and 9). These transcription factors have various roles in development, cell proliferation and differentiation [62, 63]. In agreement with GO 'artery morphogenesis' (GO:0048844) and MGI 'abnormal artery development' (MP:0003410) gene enrichments of mammal-conserved enhancers (Supplementary Tables 3 and 4) and the GO 'labyrinthine layer blood vessel development' (GO:0060716) gene enrichment of placental mammal-specific enhancers (Supplementary Table 7), ETS family members FLI1, ERG and ETV2, whose motifs are enriched in the 10-mer sets, are involved in hematopoiesis and endothelial development [64, 65]. Interestingly, AP-1 family members JUN, JunB and FOS, whose motifs are also enriched in the 10-mer sets, are involved in trophoblast cell invasion into the uterus and essential for placentation [66, 67]. This agrees with placenta-related gene enrichments of placental mammal-specific enhancers (Supplementary Tables 6 and 7) and supports placenta-related functions of these enhancers. Furthermore, 10-mers in the placental mammal-specific enhancers exhibit enriched motifs for FOXP3 (forkhead-box-protein P3). This transcription factor has been linked to preeclampsia, a pregnancy related disorder characterized by high blood pressure [68, 69].

Together, our analysis suggests that a subset of enhancers that emerged in placental mammals may have been involved in the evolution placental mammal-specific aspects of the placenta. These enhancers could serve as a starting point for more elaborate studies on the molecular basis of placenta evolution.

Summary
We generated a multiple genome alignment comprising 120 mammals and used this alignment to project human genes to 119 other mammalian genomes. To exemplify how our alignment may facilitate comparative genomics studies, we quantified the variability within ultraconserved elements and showed that placental mammal specific enhancers are significantly associated with placenta-related genes. The multiple genome alignment, sets of conserved elements, and comparative gene annotations are a valuable resource for further studies, which can be visualized in a UCSC genome browser installation at https://genome-public.pks.mpg.de/.

Materials and Methods

Phylogeny

The order level of the phylogeny is based on dos Reis *et al.* [70]. The primate phylogeny is based on Perelmann *et al.* [71]. Rodents were placed based on Fabre *et al* [72]. We based the Afrotheria phylogeny on Meredith *et al.*, Poulakakis *et al.*, and O'Leary *et al*. [73-75]. Sorex, Erinaceus, Condylura were placed based on Brace *et al*. [76]. The Carnivora phylogeny is based on Flynn *et al.* and Meredith *et al.* [73, 77]. Artiodactyla is based on O'Leary *et al.* and Ropiquet *et al*. [75, 78]. The Chiroptera phylogeny is based on Teeling *et al.* and Agnarsson *et al.* [79, 80].

Genome alignment

To compute pairwise and multiple genome alignments, we used the human hg38 assembly as the reference (Supplementary Figure 1 shows the entire workflow). We first built pairwise alignments between human and a query species using lastz and axtChain to compute co-linear alignment chains [81, 82]. To align placental mammals, we used previously-determined lastz parameters (K = 2400, L = 3000, Y = 9400, H = 2000 and the lastz default scoring matrix) that have a sufficient sensitivity to capture orthologous exons [16]. To align chimpanzee, bonobo and gorilla, we changed the lastz parameters (K=4500 and L=4500).

After building chains, we applied RepeatFiller (RRID:SCR_017414)[83], a method that performs another round of local alignment, considering unaligning regions ≤20 kb in size that are bounded by co-linear alignment blocks up- and downstream. RepeatFiller removes any repeat masking from the unaligned region and is therefore able to detect

novel alignments between repetitive regions. We have previously shown that RepeatFiller detects several megabases of aligning repetitive sequences that would be missed otherwise [83]. After RepeatFiller, we applied chainCleaner with parameters -LRfoldThreshold= 2.5 -doPairs -LRfoldThresholdPairs = 10 -maxPairDistance = 10000 -maxSuspectScore = 100000 -minBrokenChainScore = 75000 to improve alignment specificity [84]. Pairwise alignment chains were converted into alignment nets using a modified version of chainNet [82] that computes real scores of partial nets [84]. Nets were filtered using NetFilterNonNested.perl with parameters -doUCSCSynFilter -keepSynNetsWithScore 5000 -keepInvNetsWithScore 5000 [84], which applies the UCSC 'syntenic net' score thresholds (minTopScore of 300000 and minSynScore of 200000) and keeps nested nets that align to the same locus (inversions or local translocations; net type 'inv' or 'syn' according to netClass [82]) if they score ≥5000. For the Mongolian gerbil, tarsier, Malayan flying lemur, sperm whale, Przewalski's horse, Weddell seal, Malayan pangolin, Chinese pangolin, Hoffmann's two-fingered sloth, and Cape rock hyrax that have genome assemblies with a scaffold N50 ≤1,000,000 and a contig N50 ≤100,000, we just required that nets have a score ≥100,000. For marsupials and platypus, we lowered the score threshold for nets to 10,000 and kept inv or syn nets with scores ≥3000. Next, we used the filtered nets to compute a human-referenced multiple genome alignment with MULTIZ-tba [85]. Finally, to distinguish between unaligning genomic regions that are truly diverged and genomic regions that do not align because they overlap assembly gaps in the query genome [86], we post-processed the multiple genome alignment and removed all unaligning regions (e-lines in a maf block) that either overlap an assembly gap in the respective query genome(s) or are not covered by any alignment chain.

The main difference between this 120-mammal alignment and our previous 144-vertebrate alignment [16] is that the former focuses entirely on mammals and includes many new species (120 vs. 74 mammals, see Supplementary Table 1). In addition, we updated genome assemblies of twelve species that were already included in the previous alignment (species are marked in Supplementary Table 1). Finally, the 120-mammal alignment used RepeatFiller [83] to improve the completeness of alignments between repetitive regions.

Identification of conserved regions

We used msa_view to extract 4-fold degenerated codon positions based on the human RefSeq gene annotation and used PhyloFit [87] to estimate the length of all branches in the tree as substitutions per neutral site. This tree was used to detect constrained elements with PhastCons [18] and GERP++ (GERP, RRID:SCR_000563)[42]. For

running PhastCons, we used the parameters rho=0.31, expected-length=45, and target-coverage=0.3. For GERP++, we used default parameters.

Comparative gene annotation with CESAR

Genes were annotated using the CESAR gene annotation pipeline [15, 38, 39] using all protein-coding transcripts from the human ENSEMBL 96 gene annotation as input [88]. To count the number of annotated genes per species, we first extracted per locus the transcript with the longest open reading frame (ignoring all shorter overlapping transcripts) and then determined the number of unique gene symbols.

UCE divergence analysis

UCE coordinates were downloaded from UCbase2.0 [89]. We converted the coordinates of the 481 UCEs from hg19 to hg38 using liftOver. We merged UCE 208 and 209 into one UCE because they are directly adjacent. We then extracted alignments of UCEs from our 120 mammal alignment. For robustness, we only considered the 441 UCEs for which we aligned at least 110 of placental mammals over the entire length of the UCE and further removed sequences that contained assembly gaps. Next, we used a previously developed bottom-up Fitch-like parsimony approach [90] to identify alignment columns containing one or more substitutions. To account for the possibility of base errors in assemblies, we additionally identified alignment columns that have shared substitutions between at least two sister species. We used shared substitutions as a lower bound estimate for variable columns in UCE alignments. To investigate the influence of the number of considered species, we repeated this analysis for the same 441 UCEs but considered only 60 instead of all 115 non-human placental mammals (marked in Supplementary Table 1).

To investigate how variable positions are distributed within UCEs, we had to account for the different lengths of UCEs. To this end, we normalized the positions of each UCE into 100 equally sized bins. Since not all positions can be uniquely assigned to a single bin (unless the UCE length is a multiple of 100), we duplicated the value for each position in a UCE (1 for nucleotide change, 0 otherwise) 100 times and then grouped them into bins. The cumulative value of each bin was then normalized by bin size (length of the UCE) to obtain a per-UCE value for nucleotide changes at each relative position.

Analysis of FANTOM enhancers

We downloaded the coordinates of the 38,548 robust FANTOM enhancers from SlideBase [56] (http://slidebase.binf.ku.dk/human_enhancers/). Coordinates were then mapped from the human hg19 genome assembly to hg38 using liftOver. Next, we identified the most conserved 10-mers in all FANTOM enhancers using a sliding-window

approach. We then counted the number of species that were aligned with identical 10-mers per following clades: Primatomorpha, Glires, Artiodactyla, Ferae, Chiroptera, Eulipotyphla, Atlantogenata and non-placental mammals. We defined an enhancer as conserved across all mammals if at least 50% of the species in each of these clades were aligned with an identical 10-mer. For identifying placental mammal specific enhancers, we required that at least 50% of the species in each placental mammal clade were aligned with an identical 10-mer and that no sequence was aligned to the entire enhancer region for any non-placental mammal.

Enrichment analysis for placental mammal-specific enhancers

We used the GREAT webserver version 4 (Aug. 19, 2019) to test whether placental mammal-specific enhancers are enriched near genes belonging to certain functional groups [58]. We used the hg19 genome assembly coordinates and the 38,548 robust FANTOM enhancers as background [56]. We considered terms significantly enriched if they exceed a 2-fold enrichment (RegionFoldEnrich) and exhibit a corrected p-value (hypergeometric FDR Q-value) < 0.05. In addition to the enrichment analysis, we downloaded predicted placenta enhancers [59] and compared how many placental mammal-specific enhancers overlap predicted placenta enhancers. Here, we required that at least 50% of the enhancer overlaps a predicted placenta enhancer.

Motif enrichment analysis of conserved 10-mers

To identify enriched transcription factor binding motifs for mammal-conserved and placental mammal-specific enhancers, we first identified all conserved 10-mers in each enhancer set using the same criteria as described above and merged overlapping 10-mers. The human sequences of these merged 10-mers were then used as input for Analysis of Motif Enrichment (AME) from the MEME suite (RRID:SCR_001783)[60, 61]. Shuffled sequences were used as background and motifs with an e-value < 0.05 were considered as enriched.

Data Availability

The 120 mammal alignment, phylogenetic tree, conserved elements, GERP and PhastCons tracks, CESAR gene annotations for 119 non-human mammals are available for download at https://bds.mpi-cbg.de/hillerlab/120MammalAlignment/. This data can also be loaded as a trackhub [91] into the UCSC genome browser via https://bds.mpi-cbg.de/hillerlab/120MammalAlignment/Human120way/trackHub/hub.txt. Furthermore, our UCSC genome browser installation at https://genome-public.pks.mpg.de/ visualizes

all data. Snapshots of the data and code are also archived in the *GigaScience* GigaDB repository [92].

Competing interests

The authors have no competing interests.

Acknowledgment

We thank the genomics community for sequencing and assembling the genomes and the UCSC genome browser group for providing software and genome annotations. We also thank Heiko Stuckas, Thomas Lehmann and Henrike Indrischek for helpful discussions on the phylogeny and the Computer Service Facilities of the MPI-CBG and MPI-PKS for their support.

Funding

This work was supported by the Max Planck Society and the Leibniz Association (SAW-2016-SGN-2).

References

1. Miller W, Makova KD, Nekrutenko A and Hardison RC. Comparative genomics. Annu Rev Genomics Hum Genet. 2004;5:15-56. doi:10.1146/annurev.genom.5.061903.180057.
2. Alfoldi J and Lindblad-Toh K. Comparative genomics as a tool to understand evolution and disease. Genome Res. 2013;23 7:1063-8. doi:10.1101/gr.157503.113.
3. Stark A, Lin MF, Kheradpour P, Pedersen JS, Parts L, Carlson JW, et al. Discovery of functional elements in 12 Drosophila genomes using evolutionary signatures. Nature. 2007;450 7167:219-32.
4. Lindblad-Toh K, Garber M, Zuk O, Lin MF, Parker BJ, Washietl S, et al. A high-resolution map of human evolutionary constraint using 29 mammals. Nature. 2011;478 7370:476-82. doi:10.1038/nature10530.
5. Washietl S, Hofacker IL and Stadler PF. Fast and reliable prediction of noncoding RNAs. Proceedings of the National Academy of Sciences of the United States of America. 2005;102 7:2454-9. doi:10.1073/pnas.0409169102.
6. Seemann SE, Mirza AH, Hansen C, Bang-Berthelsen CH, Garde C, Christensen-Dalsgaard M, et al. The identification and functional annotation of RNA structures conserved in vertebrates. Genome Res. 2017;27 8:1371-83. doi:10.1101/gr.208652.116.
7. Hiller M, Findeiss S, Lein S, Marz M, Nickel C, Rose D, et al. Conserved introns reveal novel transcripts in Drosophila melanogaster. Genome Res. 2009;19 7:1289-300. doi:10.1101/gr.090050.108.
8. Jungreis I, Lin MF, Spokony R, Chan CS, Negre N, Victorsen A, et al. Evidence of abundant stop codon readthrough in Drosophila and other metazoa. Genome Res. 2011;21 12:2096-113. doi:10.1101/gr.119974.110.

9. Lin MF, Kheradpour P, Washietl S, Parker BJ, Pedersen JS and Kellis M. Locating protein-coding sequences under selection for additional, overlapping functions in 29 mammalian genomes. Genome Res. 2011;21 11:1916-28. doi:10.1101/gr.108753.110.
10. Alexandersson M, Cawley S and Pachter L. SLAM: cross-species gene finding and alignment with a generalized pair hidden Markov model. Genome Res. 2003;13 3:496-502. doi:10.1101/gr.424203.
11. Gross SS, Do CB, Sirota M and Batzoglou S. CONTRAST: a discriminative, phylogeny-free approach to multiple informant de novo gene prediction. Genome Biol. 2007;8 12:R269. doi:10.1186/gb-2007-8-12-r269.
12. Siepel A, Diekhans M, Brejova B, Langton L, Stevens M, Comstock CL, et al. Targeted discovery of novel human exons by comparative genomics. Genome Res. 2007;17 12:1763-73. doi:10.1101/gr.7128207.
13. Lin MF, Jungreis I and Kellis M. PhyloCSF: a comparative genomics method to distinguish protein coding and non-coding regions. Bioinformatics. 2011;27 13:i275-82. doi:10.1093/bioinformatics/btr209.
14. Konig S, Romoth LW, Gerischer L and Stanke M. Simultaneous gene finding in multiple genomes. Bioinformatics. 2016;32 22:3388-95. doi:10.1093/bioinformatics/btw494.
15. Sharma V, Elghafari A and Hiller M. Coding exon-structure aware realigner (CESAR) utilizes genome alignments for accurate comparative gene annotation. Nucleic Acids Res. 2016;44 11:e103. doi:10.1093/nar/gkw210.
16. Sharma V and Hiller M. Increased alignment sensitivity improves the usage of genome alignments for comparative gene annotation. Nucleic Acids Res. 2017;45 14:8369-77. doi:10.1093/nar/gkx554.
17. Armstrong J, Fiddes IT, Diekhans M and Paten B. Whole-Genome Alignment and Comparative Annotation. Annu Rev Anim Biosci. 2019;7:41-64. doi:10.1146/annurev-animal-020518-115005.
18. Siepel A, Bejerano G, Pedersen JS, Hinrichs AS, Hou M, Rosenbloom K, et al. Evolutionarily conserved elements in vertebrate, insect, worm, and yeast genomes. Genome Res. 2005;15 8:1034-50.
19. Cooper GM, Stone EA, Asimenos G, Green ED, Batzoglou S and Sidow A. Distribution and intensity of constraint in mammalian genomic sequence. Genome Res. 2005;15 7:901-13. doi:10.1101/gr.3577405.
20. Ponting CP and Hardison RC. What fraction of the human genome is functional? Genome Res. 2011;21 11:1769-76. doi:10.1101/gr.116814.110.
21. Woolfe A, Goodson M, Goode DK, Snell P, McEwen GK, Vavouri T, et al. Highly conserved non-coding sequences are associated with vertebrate development. PLoS Biol. 2005;3 1:e7.
22. Visel A, Prabhakar S, Akiyama JA, Shoukry M, Lewis KD, Holt A, et al. Ultraconservation identifies a small subset of extremely constrained developmental enhancers. Nat Genet. 2008;40 2:158-60. doi:10.1038/ng.2007.55.
23. McLean CY, Reno PL, Pollen AA, Bassan AI, Capellini TD, Guenther C, et al. Human-specific loss of regulatory DNA and the evolution of human-specific traits. Nature. 2011;471 7337:216-9. doi:10.1038/nature09774.
24. Hiller M, Schaar BT, Indjeian VB, Kingsley DM, Hagey LR and Bejerano G. A "forward genomics" approach links genotype to phenotype using independent phenotypic losses among related species. Cell Rep. 2012;2 4:817-23. doi:10.1016/j.celrep.2012.08.032.
25. Berger MJ, Wenger AM, Guturu H and Bejerano G. Independent erosion of conserved transcription factor binding sites points to shared hindlimb, vision and external testes loss in different mammals. Nucleic Acids Res. 2018;46 18:9299-308. doi:10.1093/nar/gky741.

26. Marcovitz A, Jia R and Bejerano G. "Reverse Genomics" Predicts Function of Human Conserved Noncoding Elements. Molecular biology and evolution. 2016;33 5:1358-69. doi:10.1093/molbev/msw001.
27. Prudent X, Parra G, Schwede P, Roscito JG and Hiller M. Controlling for Phylogenetic Relatedness and Evolutionary Rates Improves the Discovery of Associations Between Species' Phenotypic and Genomic Differences. Molecular biology and evolution. 2016;33 8:2135-50. doi:10.1093/molbev/msw098.
28. Partha R, Chauhan BK, Ferreira Z, Robinson JD, Lathrop K, Nischal KK, et al. Subterranean mammals show convergent regression in ocular genes and enhancers, along with adaptation to tunneling. eLife. 2017;6:e25884. doi:10.7554/eLife.25884.
29. Sharma V, Hecker N, Roscito JG, Foerster L, Langer BE and Hiller M. A genomics approach reveals insights into the importance of gene losses for mammalian adaptations. Nature communications. 2018;9 1:1215. doi:10.1038/s41467-018-03667-1.
30. Roscito JG, Sameith K, Parra G, Langer BE, Petzold A, Moebius C, et al. Phenotype loss is associated with widespread divergence of the gene regulatory landscape in evolution. Nature communications. 2018;9 1:4737. doi:10.1038/s41467-018-07122-z.
31. Meredith RW, Zhang G, Gilbert MT, Jarvis ED and Springer MS. Evidence for a single loss of mineralized teeth in the common avian ancestor. Science. 2014;346 6215:1254390. doi:10.1126/science.1254390.
32. Sharma V, Lehmann T, Stuckas H, Funke L and Hiller M. Loss of RXFP2 and INSL3 genes in Afrotheria shows that testicular descent is the ancestral condition in placental mammals. PLoS Biol. 2018;16 6:e2005293. doi:10.1371/journal.pbio.2005293.
33. Jebb D and Hiller M. Recurrent loss of HMGCS2 shows that ketogenesis is not essential for the evolution of large mammalian brains. eLife. 2018;7:e38906. doi:10.7554/eLife.38906.
34. Hecker N, Sharma V and Hiller M. Convergent gene losses illuminate metabolic and physiological changes in herbivores and carnivores. Proceedings of the National Academy of Sciences of the United States of America. 2019;116 8:3036-41. doi:10.1073/pnas.1818504116.
35. Eddy SR. A model of the statistical power of comparative genome sequence analysis. PLoS Biol. 2005;3 1:e10. doi:10.1371/journal.pbio.0030010.
36. Nabhan AR and Sarkar IN. The impact of taxon sampling on phylogenetic inference: a review of two decades of controversy. Brief Bioinform. 2012;13 1:122-34. doi:10.1093/bib/bbr014.
37. Thomas GW, Hahn MW and Hahn Y. The Effects of Increasing the Number of Taxa on Inferences of Molecular Convergence. Genome Biol Evol. 2017;9 1:213-21. doi:10.1093/gbe/evw306.
38. Sharma V, Schwede P and Hiller M. CESAR 2.0 substantially improves speed and accuracy of comparative gene annotation. Bioinformatics. 2017;33 24:3985-7. doi:10.1093/bioinformatics/btx527.
39. Sharma V and Hiller M. Coding Exon-Structure Aware Realigner (CESAR): Utilizing Genome Alignments for Comparative Gene Annotation. Methods in molecular biology. 2019;1962:179-91. doi:10.1007/978-1-4939-9173-0_10.
40. Hecker N, Sharma V and Hiller M. Transition to an Aquatic Habitat Permitted the Repeated Loss of the Pleiotropic KLK8 Gene in Mammals. Genome Biol Evol. 2017;9 11:3179-88. doi:10.1093/gbe/evx239.
41. Huelsmann M, Hecker N, Springer MS, Gatesy J, Sharma V and Hiller M. Genes lost during the transition from land to water in cetaceans highlight genomic changes associated with aquatic adaptations. Sci Adv. 2019;5 9:eaaw6671. doi:10.1126/sciadv.aaw6671.
42. Davydov EV, Goode DL, Sirota M, Cooper GM, Sidow A and Batzoglou S. Identifying a high fraction of the human genome to be under selective constraint using GERP++. PLoS computational biology. 2010;6 12:e1001025. doi:10.1371/journal.pcbi.1001025.

43. Ahituv N, Zhu Y, Visel A, Holt A, Afzal V, Pennacchio LA, et al. Deletion of ultraconserved elements yields viable mice. PLoS Biol. 2007;5 9:e234.
44. Dickel DE, Ypsilanti AR, Pla R, Zhu Y, Barozzi I, Mannion BJ, et al. Ultraconserved Enhancers Are Required for Normal Development. Cell. 2018;172 3:491-9 e15. doi:10.1016/j.cell.2017.12.017.
45. Schneider A, Hiller M and Buchholz F. Large-scale dissection suggests that ultraconserved elements are dispensable for mouse embryonic stem cell survival and fitness. https://wwwbiorxivorg/content/101101/683565v1. 2019.
46. Bejerano G, Pheasant M, Makunin I, Stephen S, Kent WJ, Mattick JS, et al. Ultraconserved elements in the human genome. Science. 2004;304 5675:1321-5. doi:10.1126/science.1098119.
47. Miller W, Rosenbloom K, Hardison RC, Hou M, Taylor J, Raney B, et al. 28-Way vertebrate alignment and conservation track in the UCSC Genome Browser. Genome Res. 2007;17 12:1797-808.
48. Katzman S, Kern AD, Bejerano G, Fewell G, Fulton L, Wilson RK, et al. Human genome ultraconserved elements are ultraselected. Science. 2007;317 5840:915.
49. Prescott SL, Srinivasan R, Marchetto MC, Grishina I, Narvaiza I, Selleri L, et al. Enhancer divergence and cis-regulatory evolution in the human and chimp neural crest. Cell. 2015;163 1:68-83. doi:10.1016/j.cell.2015.08.036.
50. Bourque G, Leong B, Vega VB, Chen X, Lee YL, Srinivasan KG, et al. Evolution of the mammalian transcription factor binding repertoire via transposable elements. Genome Res. 2008;18 11:1752-62. doi:10.1101/gr.080663.108.
51. Sundaram V, Cheng Y, Ma Z, Li D, Xing X, Edge P, et al. Widespread contribution of transposable elements to the innovation of gene regulatory networks. Genome Res. 2014;24 12:1963-76. doi:10.1101/gr.168872.113.
52. Carelli FN, Liechti A, Halbert J, Warnefors M and Kaessmann H. Repurposing of promoters and enhancers during mammalian evolution. Nature communications. 2018;9 1:4066. doi:10.1038/s41467-018-06544-z.
53. Emera D, Yin J, Reilly SK, Gockley J and Noonan JP. Origin and evolution of developmental enhancers in the mammalian neocortex. Proceedings of the National Academy of Sciences of the United States of America. 2016;113 19:E2617-26. doi:10.1073/pnas.1603718113.
54. Reilly SK, Yin J, Ayoub AE, Emera D, Leng J, Cotney J, et al. Evolutionary genomics. Evolutionary changes in promoter and enhancer activity during human corticogenesis. Science. 2015;347 6226:1155-9. doi:10.1126/science.1260943.
55. Lowe CB, Kellis M, Siepel A, Raney BJ, Clamp M, Salama SR, et al. Three periods of regulatory innovation during vertebrate evolution. Science. 2011;333 6045:1019-24. doi:10.1126/science.1202702.
56. Andersson R, Gebhard C, Miguel-Escalada I, Hoof I, Bornholdt J, Boyd M, et al. An atlas of active enhancers across human cell types and tissues. Nature. 2014;507 7493:455-61. doi:10.1038/nature12787.
57. Stewart AJ, Hannenhalli S and Plotkin JB. Why transcription factor binding sites are ten nucleotides long. Genetics. 2012;192 3:973-85. doi:10.1534/genetics.112.143370.
58. McLean CY, Bristor D, Hiller M, Clarke SL, Schaar BT, Lowe CB, et al. GREAT improves functional interpretation of cis-regulatory regions. Nat Biotechnol. 2010;28 5:495-501. doi:10.1038/nbt.1630.
59. Zhang J, Simonti CN and Capra JA. Genome-wide maps of distal gene regulatory enhancers active in the human placenta. PloS one. 2018;13 12:e0209611. doi:10.1371/journal.pone.0209611.
60. McLeay RC and Bailey TL. Motif Enrichment Analysis: a unified framework and an evaluation on ChIP data. BMC Bioinformatics. 2010;11:165. doi:10.1186/1471-2105-11-165.

61. Bailey TL, Johnson J, Grant CE and Noble WS. The MEME Suite. Nucleic Acids Res. 2015;43 W1:W39-49. doi:10.1093/nar/gkv416.
62. Gutierrez-Hartmann A, Duval DL and Bradford AP. ETS transcription factors in endocrine systems. Trends Endocrinol Metab. 2007;18 4:150-8. doi:10.1016/j.tem.2007.03.002.
63. Hess J, Angel P and Schorpp-Kistner M. AP-1 subunits: quarrel and harmony among siblings. Journal of cell science. 2004;117 Pt 25:5965-73. doi:10.1242/jcs.01589.
64. Liu F, Walmsley M, Rodaway A and Patient R. Fli1 acts at the top of the transcriptional network driving blood and endothelial development. Current biology : CB. 2008;18 16:1234-40. doi:10.1016/j.cub.2008.07.048.
65. Morita R, Suzuki M, Kasahara H, Shimizu N, Shichita T, Sekiya T, et al. ETS transcription factor ETV2 directly converts human fibroblasts into functional endothelial cells. Proceedings of the National Academy of Sciences of the United States of America. 2015;112 1:160-5. doi:10.1073/pnas.1413234112.
66. Peng B, Zhu H, Ma L, Wang YL, Klausen C and Leung PC. AP-1 Transcription Factors c-FOS and c-JUN Mediate GnRH-Induced Cadherin-11 Expression and Trophoblast Cell Invasion. Endocrinology. 2015;156 6:2269-77. doi:10.1210/en.2014-1871.
67. Schorpp-Kistner M, Wang ZQ, Angel P and Wagner EF. JunB is essential for mammalian placentation. EMBO J. 1999;18 4:934-48. doi:10.1093/emboj/18.4.934.
68. Norouzian M, Rahimzadeh M, Rajaee M, Arabpour F and Naderi N. FoxP3 gene promoter polymorphism affects susceptibility to preeclampsia. Hum Immunol. 2016;77 12:1232-8. doi:10.1016/j.humimm.2016.09.001.
69. Chen X, Gan T, Liao Z, Chen S and Xiao J. Foxp3 (-/ATT) polymorphism contributes to the susceptibility of preeclampsia. PloS one. 2013;8 4:e59696. doi:10.1371/journal.pone.0059696.
70. dos Reis M, Inoue J, Hasegawa M, Asher RJ, Donoghue PC and Yang Z. Phylogenomic datasets provide both precision and accuracy in estimating the timescale of placental mammal phylogeny. Proceedings Biological sciences / The Royal Society. 2012;279 1742:3491-500. doi:10.1098/rspb.2012.0683.
71. Perelman P, Johnson WE, Roos C, Seuanez HN, Horvath JE, Moreira MA, et al. A molecular phylogeny of living primates. PLoS Genet. 2011;7 3:e1001342. doi:10.1371/journal.pgen.1001342.
72. Fabre PH, Hautier L, Dimitrov D and Douzery EJ. A glimpse on the pattern of rodent diversification: a phylogenetic approach. BMC Evol Biol. 2012;12:88. doi:10.1186/1471-2148-12-88.
73. Meredith RW, Janecka JE, Gatesy J, Ryder OA, Fisher CA, Teeling EC, et al. Impacts of the Cretaceous Terrestrial Revolution and KPg extinction on mammal diversification. Science. 2011;334 6055:521-4. doi:10.1126/science.1211028.
74. Poulakakis N and Stamatakis A. Recapitulating the evolution of Afrotheria: 57 genes and rare genomic changes (RGCs) consolidate their history. Systematics and Biodiversity. 2010;8 3:395-408. doi:10.1080/14772000.2010.484436.
75. O'Leary MA, Bloch JI, Flynn JJ, Gaudin TJ, Giallombardo A, Giannini NP, et al. The placental mammal ancestor and the post-K-Pg radiation of placentals. Science. 2013;339 6120:662-7. doi:10.1126/science.1229237.
76. Brace S, Thomas JA, Dalen L, Burger J, MacPhee RD, Barnes I, et al. Evolutionary History of the Nesophontidae, the Last Unplaced Recent Mammal Family. Molecular biology and evolution. 2016;33 12:3095-103. doi:10.1093/molbev/msw186.
77. Flynn JJ, Finarelli JA, Zehr S, Hsu J and Nedbal MA. Molecular phylogeny of the carnivora (mammalia): assessing the impact of increased sampling on resolving enigmatic relationships. Systematic biology. 2005;54 2:317-37. doi:10.1080/10635150590923326.

78. Ropiquet A and Hassanin A. Molecular phylogeny of caprines (Bovidae, Antilopinae): the question of their origin and diversification during the Miocene. Journal of Zoological Systematics and Evolutionary Research. 2005;43 1:49-60. doi:doi:10.1111/j.1439-0469.2004.00290.x.
79. Teeling EC, Springer MS, Madsen O, Bates P, O'Brien S J and Murphy WJ. A molecular phylogeny for bats illuminates biogeography and the fossil record. Science. 2005;307 5709:580-4. doi:10.1126/science.1105113.
80. Agnarsson I, Zambrana-Torrelio CM, Flores-Saldana NP and May-Collado LJ. A time-calibrated species-level phylogeny of bats (Chiroptera, Mammalia). PLoS Curr. 2011;3:RRN1212. doi:10.1371/currents.RRN1212.
81. Harris RS. *Improved pairwise alignment of genomic DNA*. The Pennsylvania State University, 2007. PhD Thesis.
82. Kent WJ, Baertsch R, Hinrichs A, Miller W and Haussler D. Evolution's cauldron: duplication, deletion, and rearrangement in the mouse and human genomes. Proceedings of the National Academy of Sciences of the United States of America. 2003;100 20:11484-9. doi:10.1073/pnas.1932072100.
83. Osipova E, Hecker N and Hiller M. RepeatFiller newly identifies megabases of aligning repetitive sequences and improves annotations of conserved non-exonic elements. Gigascience. 2019;8 11 doi:10.1093/gigascience/giz132.
84. Suarez HG, Langer BE, Ladde P and Hiller M. chainCleaner improves genome alignment specificity and sensitivity. Bioinformatics. 2017;33 11:1596-603. doi:10.1093/bioinformatics/btx024.
85. Blanchette M, Kent WJ, Riemer C, Elnitski L, Smit AF, Roskin KM, et al. Aligning multiple genomic sequences with the threaded blockset aligner. Genome Res. 2004;14 4:708-15. doi:10.1101/gr.1933104.
86. Hiller M, Schaar BT and Bejerano G. Hundreds of conserved non-coding genomic regions are independently lost in mammals. Nucleic Acids Res. 2012;40 22:11463-76. doi:10.1093/nar/gks905.
87. Hubisz MJ, Pollard KS and Siepel A. PHAST and RPHAST: phylogenetic analysis with space/time models. Brief Bioinform. 2011;12 1:41-51. doi:10.1093/bib/bbq072.
88. Zerbino DR, Achuthan P, Akanni W, Amode MR, Barrell D, Bhai J, et al. Ensembl 2018. Nucleic Acids Res. 2018;46 D1:D754-D61. doi:10.1093/nar/gkx1098.
89. Lomonaco V, Martoglia R, Mandreoli F, Anderlucci L, Emmett W, Bicciato S, et al. UCbase 2.0: ultraconserved sequences database (2014 update). Database : the journal of biological databases and curation. 2014;2014 doi:10.1093/database/bau062.
90. Hecker N, Seemann SE, Silahtaroglu A, Ruzzo WL and Gorodkin J. Associating transcription factors and conserved RNA structures with gene regulation in the human brain. Scientific reports. 2017;7 1:5776. doi:10.1038/s41598-017-06200-4.
91. Raney BJ, Dreszer TR, Barber GP, Clawson H, Fujita PA, Wang T, et al. Track data hubs enable visualization of user-defined genome-wide annotations on the UCSC Genome Browser. Bioinformatics. 2014;30 7:1003-5. doi:10.1093/bioinformatics/btt637.
92. Hecker N; Hiller M (2019): Supporting data for "A genome alignment of 120 mammals highlights ultraconserved element variability and placenta associated enhancers" GigaScience Database. http://dx.doi.org/10.5524/100685

Figures

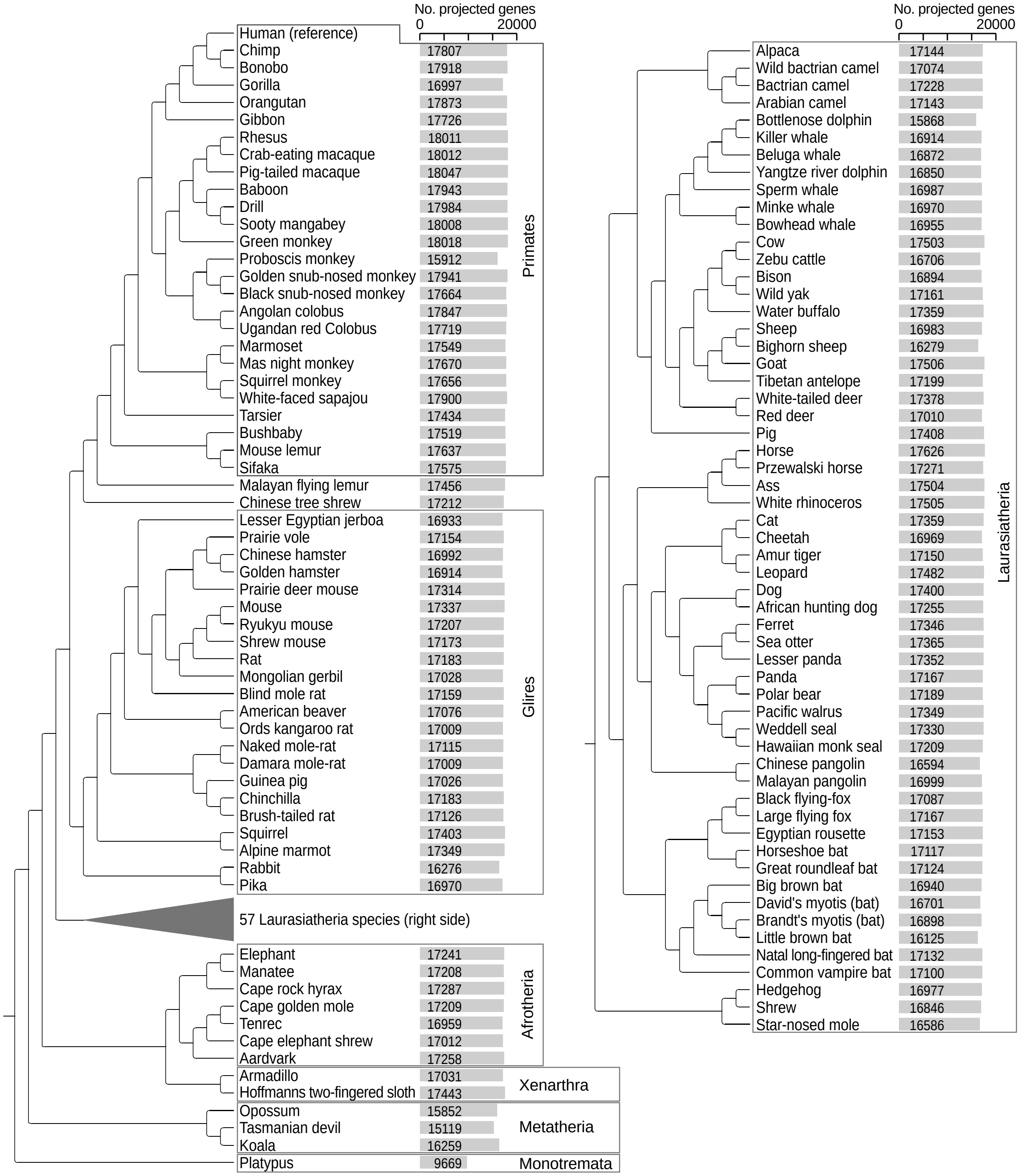

Figure 1: Phylogeny of 120 mammals included in our alignment and number of annotated genes.

Bars visualize the number of human genes for which we projected at least one intact exon. Major groups of mammals are indicated. The 57 Laurasiatheria species are shown on the right side for space reasons.

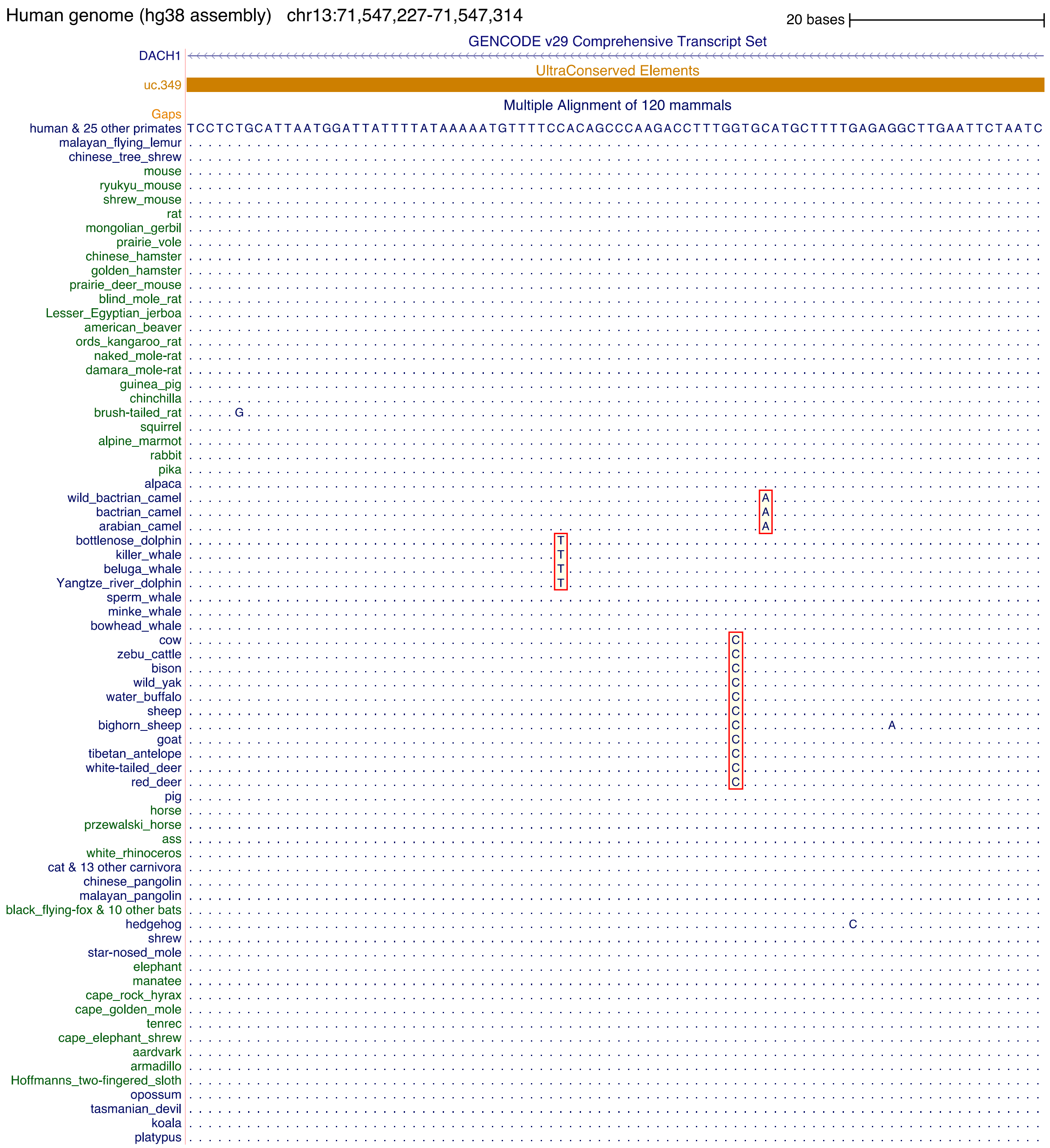

Figure 2: Example of a sequence alignment of 120 mammals showing an 88 bp region inside a UCE.

This UCE is located in an intron of the *DACH1* gene, which encodes a transcription factor important for development. Dots in the 120-mammal alignment refer to bases that are identical to the human genome. For space reasons, 25 primates, 13 carnivora and 10 bats that all have identical sequence to human are not shown. The alignment of this ultraconserved region shows that most columns are identical across all 120 mammals but also reveals a few substitutions. Some these substitutions are species-specific and may

be attributed to base errors in the assembly. Other substitutions are shared among independently sequenced genomes of related species (red boxes), which makes base errors very unlikely. We used shared substitutions to calculate a lower bound for the percentage of UCE positions that can vary across placental mammals. We used both shared and species-specific substitutions to calculate an upper bound for this percentage.

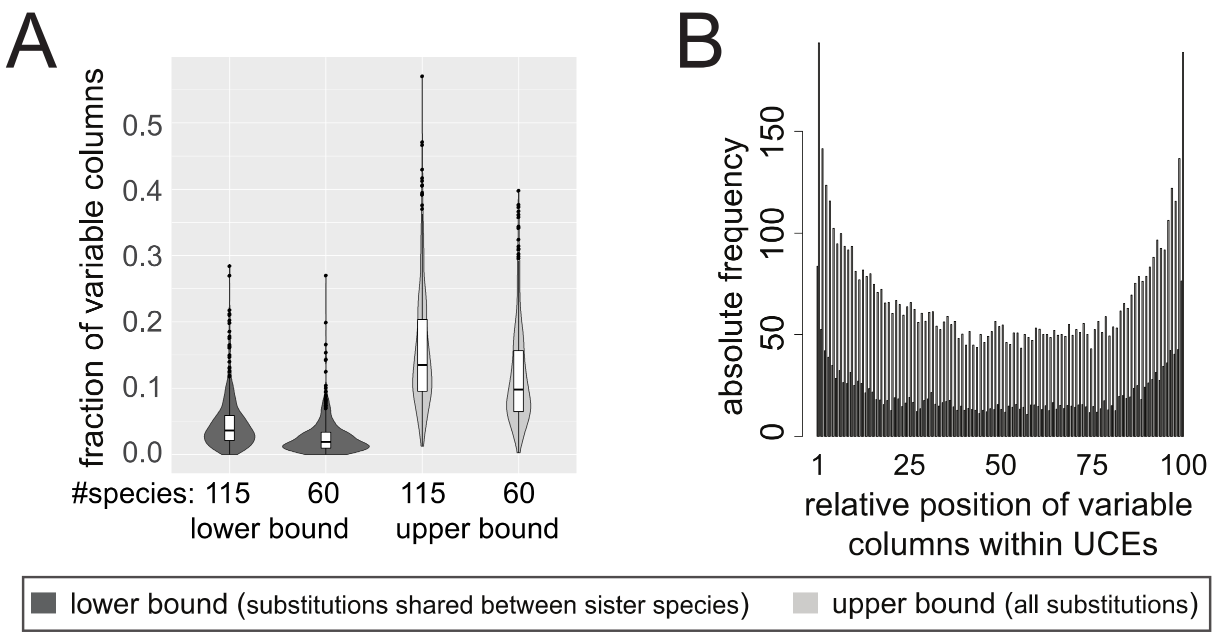

Figure 3: Variability of UCEs across placental mammals.
For each alignment position in the 441 UCEs for which at least 110 placental mammals had aligning sequence in our genome alignment, we examined whether positions in the UCE are identical or were substituted at least once across the 115 non-human placental mammals.
(A) Violin and box plots show the distribution of the fraction of variable positions per UCE across placental mammals. In addition to considering all 115 non-human placental mammals, we also determined the fraction of variable positions per UCE considering only 60 non-human placental mammals. This illustrates that analyzing fewer species would underestimate UCE variability. (B) Bar plots show the number of substitutions observed in UCEs with respect to their relative position in UCEs. UCEs were divided into 100 equally sized bins. Both upper and lower bounds show that UCEs are more variable at their flanks than in their center.

Figure 1

Click here to access/download;Figure;Figure1.pdf

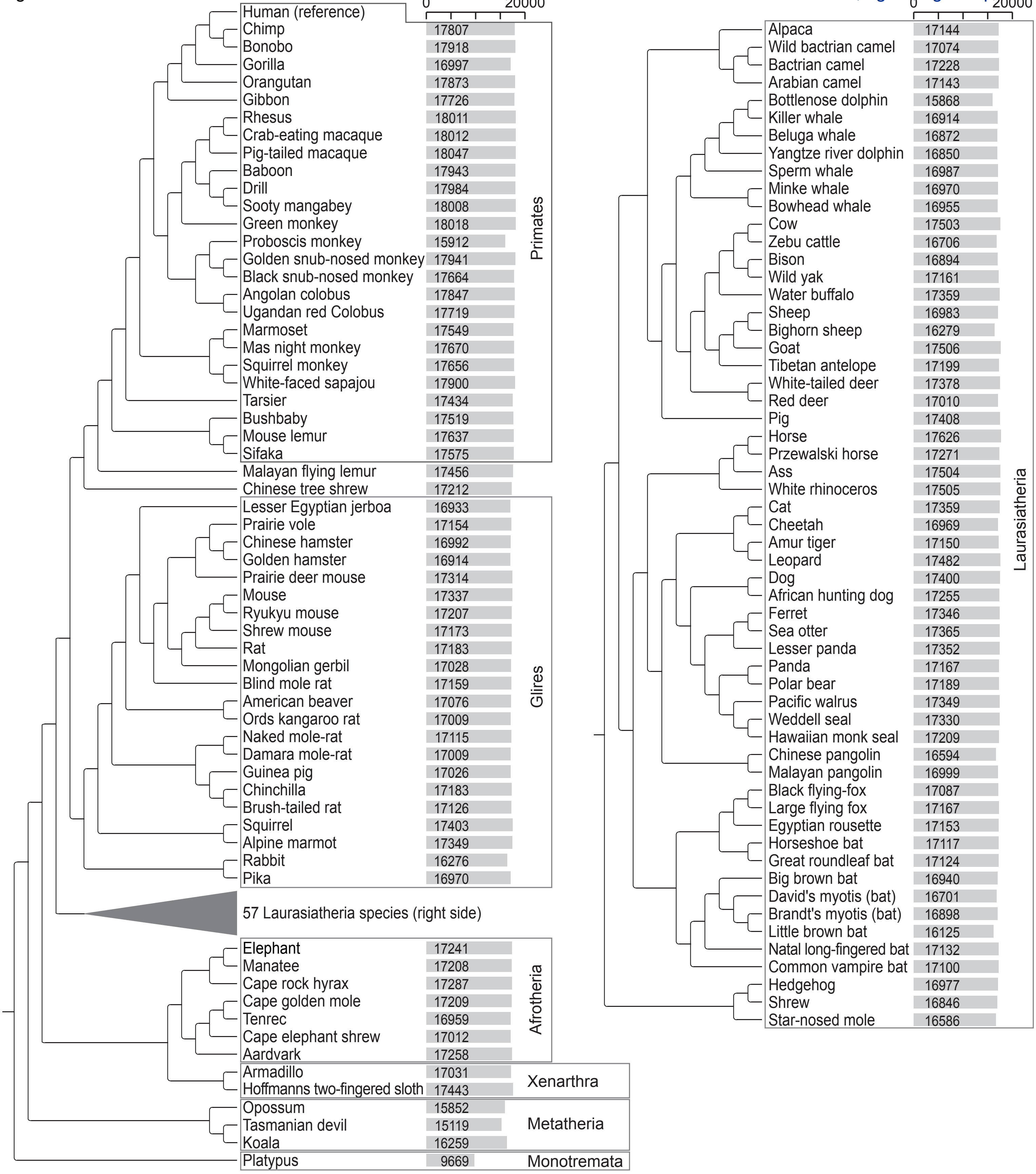

Figure 2

Click here to access/download;Figure;Figure2.pdf

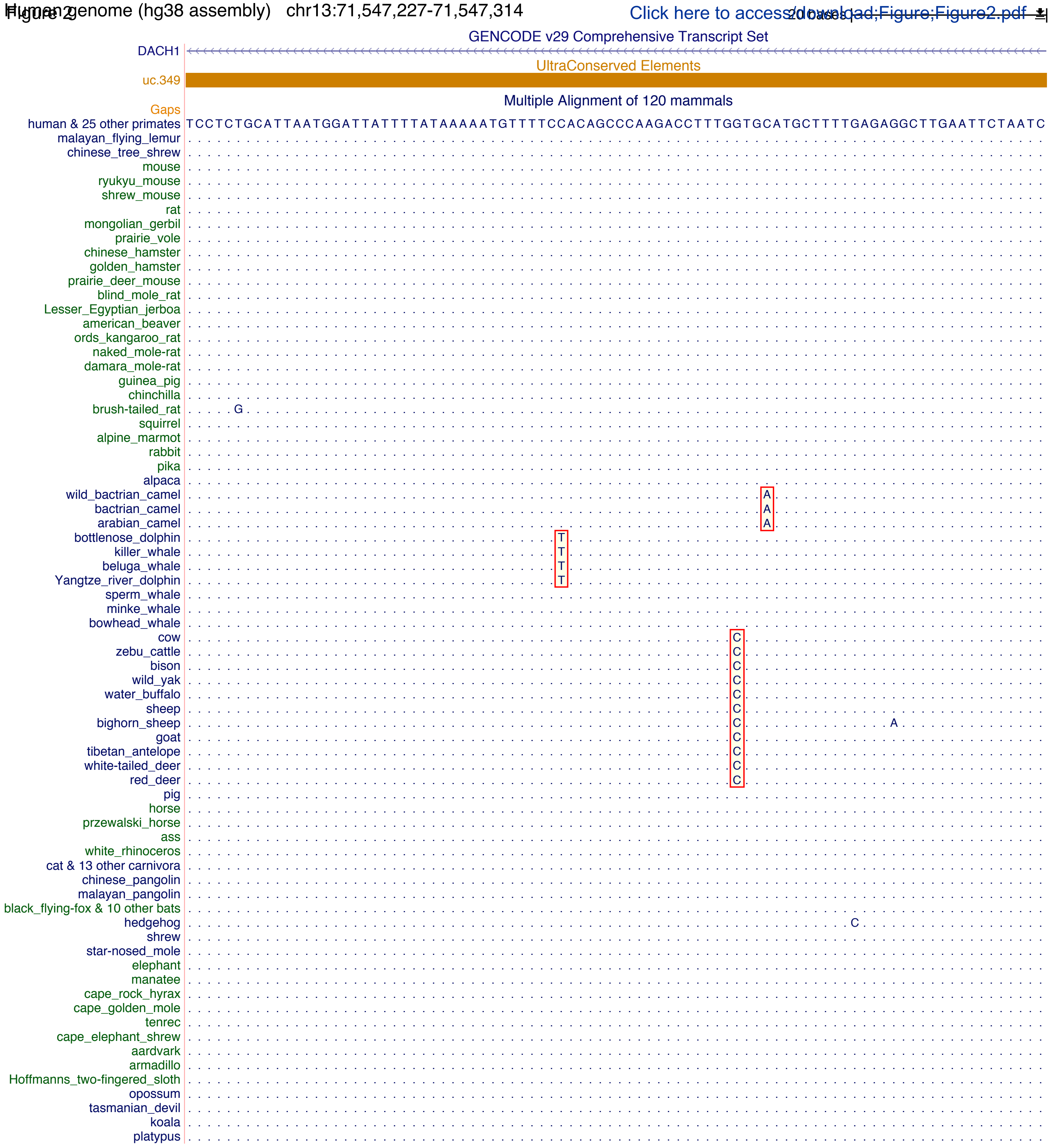

Figure 3

Click here to access/download;Figure;Figure3.

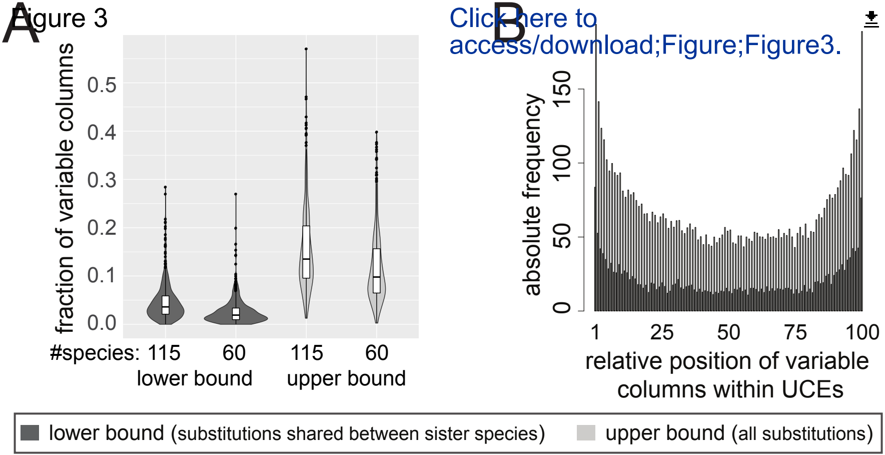

Supplementary Figures

Click here to access/download
Supplementary Material
Supplement.pdf

Supplementary Tables

Click here to access/download
Supplementary Material
SupplementaryTables.xlsx

Click here to access/download
Supplementary Material
PointByPointResponse.docx
