# Supplementary material for: A genome alignment of 120 mammals highlights ultraconserved element variability and placenta-associated enhancers
Source: Gigascience. 2020 Jan 3;9(1):giz159. doi: 10.1093/gigascience/giz159 (PMC6941714; doi:10.1093/gigascience/giz159)
Supplement: giz159_Supplemental_Files [file giz159_supplemental_files.zip › Supplement.pdf]

Supplementary Material for
A genome alignment of 120 mammals highlights ultraconserved element variability and placenta associated enhancers

Nikolai Hecker [1,2,3] and Michael Hiller [1,2,3*]

[1]Max Planck Institute of Molecular Cell Biology and Genetics, Dresden, Germany
[2]Max Planck Institute for the Physics of Complex Systems, Dresden, Germany
[3]Center for Systems Biology Dresden, Germany

*To whom correspondence should be addressed:
Michael Hiller
Computational Biology and Evolutionary Genomics, Max Planck Institute of Molecular Cell Biology and Genetics & Max Planck Institute for the Physics of Complex Systems, Dresden, Germany.
Tel: +49 351 210 2781
Fax: +49 351 210 1209
Email: hiller@mpi-cbg.de

The Supplementary Material contains
- Figures 1 – 2
- Supplementary References.

Supplementary Tables 1-9 are provided as sheets in a separate Excel file.

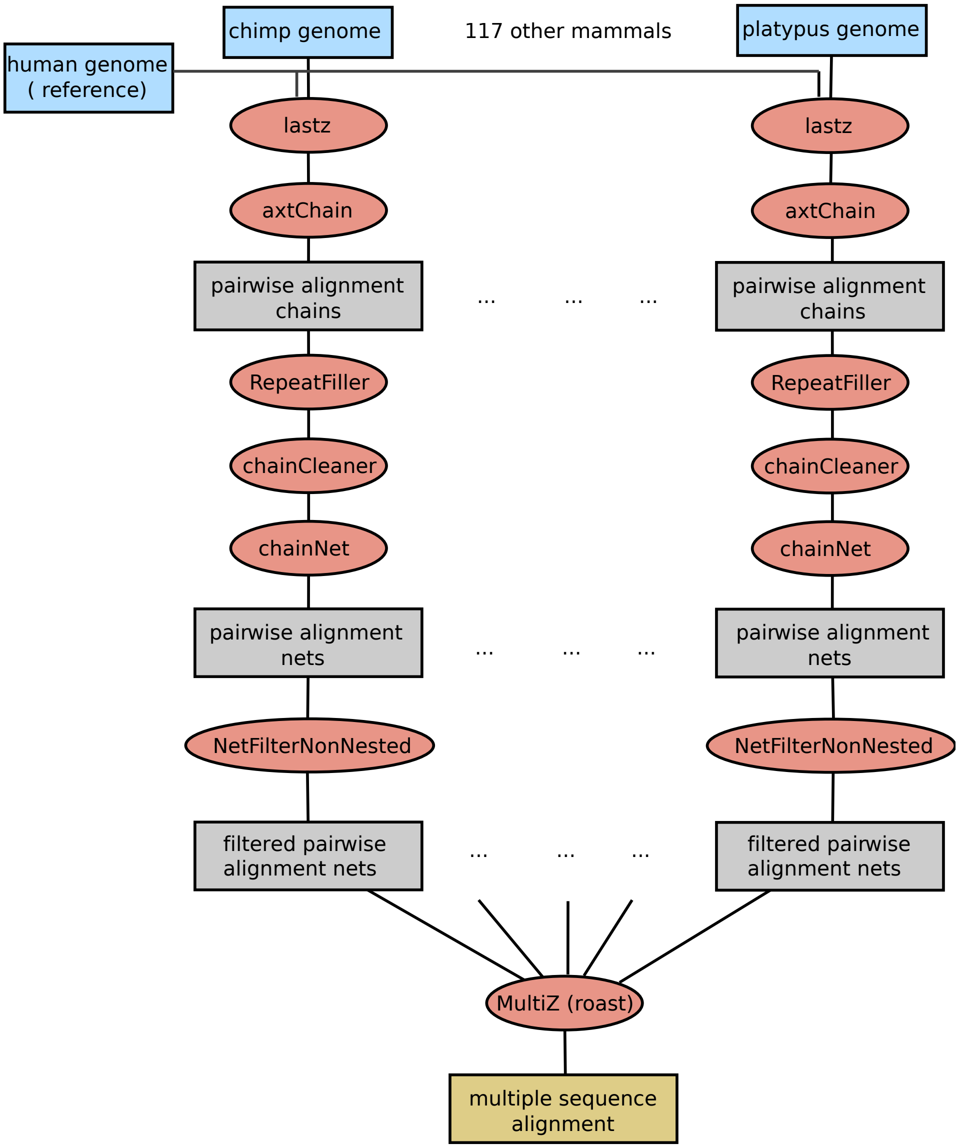

Supplementary Figure 1: Genome alignment workflow.

Input genome assemblies are indicated by light-blue rectangles, intermediate data (chains and nets) by gray rectangles, and the resulting multiple sequence alignment by a golden rectangle. Red ellipses depict the tools that were used for computing the alignments: *lastz* is used for computing local pairwise alignments between the human genome assembly and each of the 119 other mammal genome assemblies; *axtChain* [1] extracts co-linear local alignments that occur in the same order and same strand on a reference and a query chromosome and builds pairwise co-linear alignment chains; *RepeatFiller* [2] and *chainCleaner* [3] improve the pairwise alignment chains; *chainNet* [1] generates pairwise alignment nets by building a hierarchical collection of chains or parts of chains such that each locus in the reference is covered by at least one alignment to the query; *NetFilterNonNested* [3] removes low scoring and non-syntenic parts of nets in a non-nested fashion to generate the final filtered pairwise alignments. These 119 pairwise alignments are the input for *MultiZ* [4], which computes the multiple sequence alignment of 120 mammals.

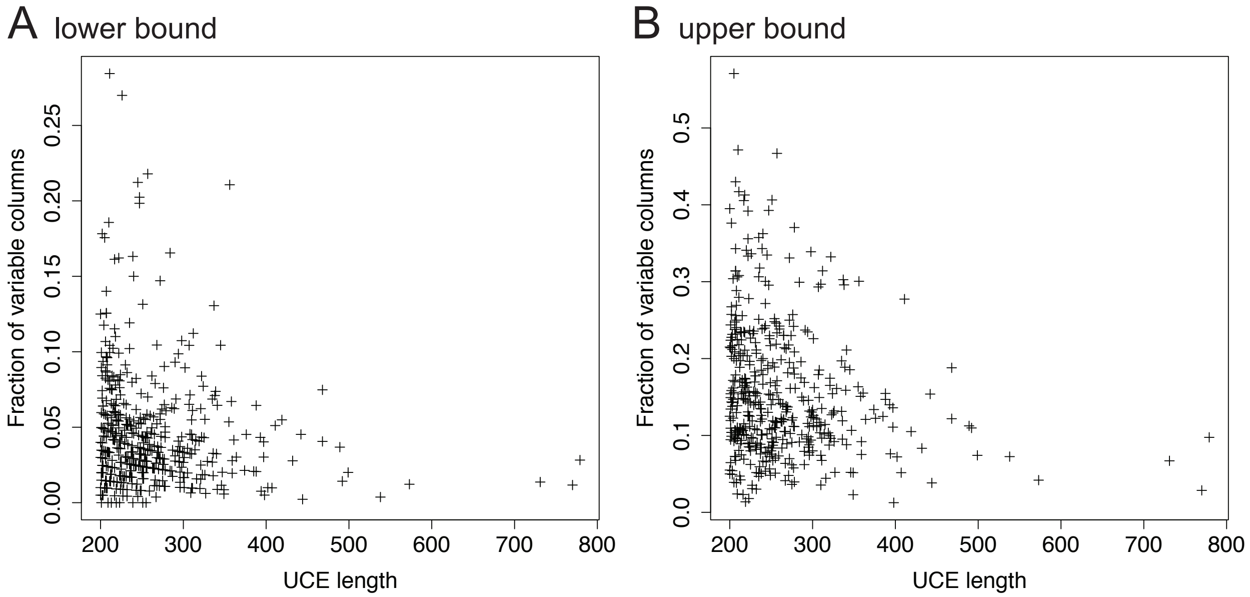

Supplementary Figure 2: Relationship between the variability and length of UCEs. Scatter plots show that there is a weak negative correlation between the fraction of variable columns and the length of UCEs.

(A) For the lower bound value for the fraction of variable columns (only considering shared substitutions), we obtain Kendall's tau of -0.11 with p-value < 10^{-3}.

(B) For the upper bound value for the fraction of variable columns (considering all substitutions), we obtain Kendall's tau of -0.12 with p-value < 10^{-3}.

This indicates that larger UCEs tend to be slightly less variable than smaller UCEs. Kendall's tau is preferred over Spearman's rank correlation if the data contains ties.

Supplementary References

1. Kent WJ, Baertsch R, Hinrichs A, Miller W and Haussler D. Evolution's cauldron: duplication, deletion, and rearrangement in the mouse and human genomes. Proceedings of the National Academy of Sciences of the United States of America. 2003;100 20:11484-9. doi:10.1073/pnas.1932072100.
2. Osipova E, Hecker N and Hiller M. RepeatFiller newly identifies megabases of aligning repetitive sequences and improves annotations of conserved non-exonic elements. Gigascience. 2019;8 11 doi:10.1093/gigascience/giz132.
3. Suarez HG, Langer BE, Ladde P and Hiller M. chainCleaner improves genome alignment specificity and sensitivity. Bioinformatics. 2017;33 11:1596-603. doi:10.1093/bioinformatics/btx024.
4. Blanchette M, Kent WJ, Riemer C, Elnitski L, Smit AF, Roskin KM, et al. Aligning multiple genomic sequences with the threaded blockset aligner. Genome Res. 2004;14 4:708-15. doi:10.1101/gr.1933104.
