# Supplementary material for: A genome alignment of 120 mammals highlights ultraconserved element variability and placenta-associated enhancers
Source: Gigascience. 2020 Jan 3;9(1):giz159. doi: 10.1093/gigascience/giz159 (PMC6941714; doi:10.1093/gigascience/giz159)
Supplement: giz159_Response_to_Reviewer_Comments_Original_Submission [file giz159_response_to_reviewer_comments_original_submission.pdf]

Author's Response To Reviewer Comments

Close

Nov 29th, 2019
Dear Dr. Edmunds,

Thank you very much for considering a revised version of our manuscript "A genome alignment of 120 mammals highlights ultraconserved element variability and placenta associated enhancers".

We would like to thank all both reviewers for their insightful comments. We have now addressed all points raised and revised the manuscript accordingly. In particular, we have
• performed an analysis of enriched transcription factor binding motifs, which revealed enrichments of factors that are likely relevant for vasculature and placenta-related gene enrichments associated with the respective enhancer sets (new Supplementary Tables 8 and 9),
• analyzed a genome alignment where the number of species was subsampled to 50%, which showed that a reduced number of species would underestimate UCE variability (updated Figure 3A),
• show the UCE length is negatively correlated with variability (new Supplementary Figure 2),
• made several text changes to clarify the definitions of conserved elements and ultraconserved elements, and provide more background for the methods used to detect conserved genomic regions,
• added a workflow of how the multiple genome alignment was generated (new Supplementary Figure 1),
• and added alignments of the UCE sequences in fasta format.

To make it really easy to use and browse our whole genome alignment of 120 mammals and the gene annotations of 119 non-human species, we have now added all data to a public UCSC genome browser installation that is freely accessible at https://genome-public.pks.mpg.de/.

We would kindly like to ask that the UCE alignments in FASTA format (https://bds.mpi-cbg.de/hillerlab/120MammalAlignment/Human120way/data/uce/) will be incorporated into the GigaDB entry associated with our manuscript.

Text changes are highlighted in red font in the manuscript. Our point-by-point response to the comments raised by the reviewers is uploaded as a separate Word document (labeled as Supplement, since there is no other appropriate category).

We have no new software to register.

We hope that our revised manuscript is now acceptable for publication. We look forward to hearing from you.

Sincerely,
Michael Hiller

Close
