# Supplementary material for: A genome alignment of 120 mammals highlights ultraconserved element variability and placenta-associated enhancers
Source: Gigascience. 2020 Jan 3;9(1):giz159. doi: 10.1093/gigascience/giz159 (PMC6941714; doi:10.1093/gigascience/giz159)
Supplement: giz159_Reviewer_1_Report_Original_Submission — Hsin-Nan Lin -- 10/30/2019 Reviewed [file giz159_reviewer_1_report_original_submission.pdf]

Reviewer Report

Title: A genome alignment of 120 mammals highlights ultraconserved element variability and placenta associated enhancers

Version: Original Submission **Date:** 10/30/2019

Reviewer name: Hsin-Nan Lin

Reviewer Comments to Author:

The authors proposed a workflow to analyze the genome alignment of 120 mammals and demonstrated the utility of the alignment by two exemplary analyses. The authors quantified the divergence in ultraconserved elements and identified placental mammal specific enhancers. The authors demonstrated the applications of genome alignment by using Bioinformatics tools. However, I have some concerns.

Firstly, the analysis methods looked complicated. Though the authors have described the analysis method and the tools they used in each step, it still seems difficult to understand. The authors should provide a more detailed workflow to illustrate the interactions between those tools. The input and output of each tool should be explicitly described in each step. The authors should also describe which parts required manual manipulation. I'd like to see the multiple sequence alignment of the 480 UCEs, however, the MAF files contained too many uninformative alignments. Most of the alignments are short and not conserved.

Secondly, the authors claimed the number of species included in the genome alignment is a key factor of affecting the power of comparative analyses, thus the authors generated a genome alignment of 120 mammals. However, I was wondering how the number of species affect the power of comparative analyses. The authors should provide another analyze that includes fewer number of species. For example, the authors could generate another genome alignment of mammals with half of species in each mammalian order.

Thirdly, the authors identified huge number of conserved elements, however, the definition of conserved elements is not clear. The definition of an ultraconserved element is an interval of at least 200bp with 100% identity [45]. I was wondering what the difference of definitions between conserved elements and ultraconserved elements is. According to [18], conserved elements are defined as intervals of at least 100bp with >70% identity. However, the author used PhastCons to identify conserved regions with expected-length=45 and target-coverage=0.3. It seems a bit confusing about the definition of conserved elements.

Fourthly, the author did not explain what an intact exon alignment is. It is estimated there were, on average, 5.48 exons per gene. How did the author define a human gene has intact exon alignments in mammals? If a human gene contains 10 exon and only one of its exon is aligned with chick gene, will it be counted or not?

Lastly, the authors should provide more comprehensive observations about the genome alignment of 120 mammals. For example, the average of sequence identity or the average number of conserved elements in each mammalian order.

minor: Human UCE sequences are conserved in the chicken genome with an average of 95% identity rather than 96%[45].

Level of Interest

Please indicate how interesting you found the manuscript: Choose an item.

Quality of Written English

Please indicate the quality of language in the manuscript: Choose an item.

Declaration of Competing Interests

Please complete a declaration of competing interests, considering the following questions:

- Have you in the past five years received reimbursements, fees, funding, or salary from an organisation that may in any way gain or lose financially from the publication of this manuscript, either now or in the future?
- Do you hold any stocks or shares in an organisation that may in any way gain or lose financially from the publication of this manuscript, either now or in the future?
- Do you hold or are you currently applying for any patents relating to the content of the manuscript?
- Have you received reimbursements, fees, funding, or salary from an organization that holds or has applied for patents relating to the content of the manuscript?
- Do you have any other financial competing interests?
- Do you have any non-financial competing interests in relation to this paper?

If you can answer no to all of the above, write 'I declare that I have no competing interests' below. If your reply is yes to any, please give details below.

I declare that I have no competing interests.

I agree to the open peer review policy of the journal. I understand that my name will be included on my report to the authors and, if the manuscript is accepted for publication, my named report including any attachments I upload will be posted on the website along with the authors' responses. I agree for my report to be made available under an Open Access Creative Commons CC-BY license (http://creativecommons.org/licenses/by/4.0/). I understand that any comments which I do not wish to be included in my named report can be included as confidential comments to the editors, which will not be published.

Choose an item.

To further support our reviewers, we have joined with Publons, where you can gain additional credit to further highlight your hard work (see: https://publons.com/journal/530/gigascience). On publication of this paper, your review will be automatically added to Publons, you can then choose whether or not to claim your Publons credit. I understand this statement.

Yes Choose an item.
