# Supplementary material for: A genome alignment of 120 mammals highlights ultraconserved element variability and placenta-associated enhancers
Source: Gigascience. 2020 Jan 3;9(1):giz159. doi: 10.1093/gigascience/giz159 (PMC6941714; doi:10.1093/gigascience/giz159)
Supplement: giz159_Reviewer_2_Report_Original_Submission — Quan Nguyen -- 11/6/2019 Reviewed [file giz159_reviewer_2_report_original_submission.pdf]

Reviewer Report

Title: A genome alignment of 120 mammals highlights ultraconserved element variability and placenta associated enhancers

Version: Original Submission **Date:** 11/6/2019

Reviewer name: Quan Nguyen

Reviewer Comments to Author:

The authors present a valuable resource for comparative genomics analysis and demonstrate important insights into the sequences of ultraconserved elements and conserved enhancers.
My minor comments and suggestions are below:
The definition of conserved placental mammal specific enhancers is based on the presence of at least one shared 10-mer across all placental mammal clades, assumming that the 10-mer represents the typical size for transcription factor (TF) binding sites. It would be interesting to predict which TF binding sites are enriched in all of those 10-mers identified for 1820 enhancers and if these TFs are related to placenta-related genes. Results from this analysis would serve as orthogonal validation of the GREAT enrichment analysis.
Among the FANTOM conserved enhancers, are there enhancers with experimental validation to be active enhancers for human and mouse as reported in other databases such as the VISTA database?
What improvements or new features does the Human120Way alignment have compared to other publicly available enhancers, for example in comparison with the multiple alignment of 144 vertebrate genomes, which were also contains the mapping of human genes to other 133 species using CESAR (Sharma and Hiller, 2017)?
Regarding Supplementary Table S2, it appears the variability measured by lower bounds and the upper bounds is inversely proportional to the length, i.e. longer regions have lower values. It will be useful to describe in more details, how the UCEs were determined and explain why their lengths are different. To make the bounds comparable between regions, the authors may consider weighting the variability by length for each region.
Very minor: Use a full name for CESAR (Coding exon-structure aware realigner) when it is first mentioned

Level of Interest

Please indicate how interesting you found the manuscript: Choose an item.

Quality of Written English

Please indicate the quality of language in the manuscript: Choose an item.

Declaration of Competing Interests

Please complete a declaration of competing interests, considering the following questions:

- Have you in the past five years received reimbursements, fees, funding, or salary from an organisation that may in any way gain or lose financially from the publication of this manuscript, either now or in the future?
- Do you hold any stocks or shares in an organisation that may in any way gain or lose financially from the publication of this manuscript, either now or in the future?
- Do you hold or are you currently applying for any patents relating to the content of the manuscript?
- Have you received reimbursements, fees, funding, or salary from an organization that holds or has applied for patents relating to the content of the manuscript?
- Do you have any other financial competing interests?
- Do you have any non-financial competing interests in relation to this paper?

If you can answer no to all of the above, write 'I declare that I have no competing interests' below. If your reply is yes to any, please give details below.

I declare that I have no competing interests

I agree to the open peer review policy of the journal. I understand that my name will be included on my report to the authors and, if the manuscript is accepted for publication, my named report including any attachments I upload will be posted on the website along with the authors' responses. I agree for my report to be made available under an Open Access Creative Commons CC-BY license (http://creativecommons.org/licenses/by/4.0/). I understand that any comments which I do not wish to be included in my named report can be included as confidential comments to the editors, which will not be published.

Choose an item.

To further support our reviewers, we have joined with Publons, where you can gain additional credit to further highlight your hard work (see: https://publons.com/journal/530/gigascience). On publication of this paper, your review will be automatically added to Publons, you can then choose whether or not to claim your Publons credit. I understand this statement.

Yes Choose an item.
