# Supplementary material for: A genome alignment of 120 mammals highlights ultraconserved element variability and placenta-associated enhancers
Source: Gigascience. 2020 Jan 3;9(1):giz159. doi: 10.1093/gigascience/giz159 (PMC6941714; doi:10.1093/gigascience/giz159)
Supplement: giz159_Supplemental_Files [file giz159_supplemental_files.zip › PointByPointResponse.docx]

**Point by point response**

**New addition**

Although not requested by the reviewers, we have now added our whole genome alignment of 120 mammals and the gene annotations of 119 non-human species to a public UCSC genome browser installation that is freely accessible at <https://genome-public.pks.mpg.de/>. There is a link to the human 120-mammal alignment from the front page. This will facilitate using the resources presented in this manuscript by the community.

In addition, all data is still available for download and for loading as a UCSC track hub, as before.

**Reviewer #1:**

The authors proposed a workflow to analyze the genome alignment of 120 mammals and demonstrated the utility of the alignment by two exemplary analyses. The authors quantified the divergence in ultraconserved elements and identified placental mammal specific enhancers. The authors demonstrated the applications of genome alignment by using Bioinformatics tools. However, I have some concerns.

We thank the reviewer for the comments and think that our changes substantially improved the clarity our manuscript.

The main purpose of this manuscript is to provide a rich data set consisting of a multiple whole genome alignment and annotated genes and conserved elements. We then used two case studies to show how such an alignment can be used for comparative studies. To enhance the clarity of our manuscript and to make clear that we generated a whole genome alignment of 120 mammals, we added a new paragraph at the beginning of the results section:

**“Generating a multiple genome alignment of 120 mammals**

To compute a comprehensive multiple genome alignment of mammals, we used human as the reference species and aligned 119 non-human mammals that have genome assemblies with a scaffold N50 value of at least 100,000 (Supplementary Table 1). The phylogeny of these 120 species is shown in Figure 1. The workflow and methods used to compute the alignment are shown in Supplementary Figure 1.”

Firstly, the analysis methods looked complicated. Though the authors have described the analysis method and the tools they used in each step, it still seems difficult to understand. The authors should provide a more detailed workflow to illustrate the interactions between those tools. The input and output of each tool should be explicitly described in each step. The authors should also describe which parts required manual manipulation.

The reviewer is correct that generating a multiple whole-genome alignment requires a number of steps and tools. As suggested, to provide an overview of the workflow that we employed to generate the 120 mammal whole-genome alignment, we have added a new Supplementary Figure 1 that shows all tools with their inputs and outputs and how they are connected.

**Supplementary Figure 1:** Genome alignment workflow.

Input genome assemblies are indicated by light-blue rectangles, intermediate data (chains and nets) by gray rectangles, and the resulting multiple sequence alignment by a golden rectangle. Red ellipses depict the tools that were used for computing the alignments: *lastz* is used for computing local pairwise alignments between the human genome assembly and each of the 119 other mammal genome assemblies; *axtChain* [1] extracts co-linear local alignments that occur in the same order and same strand on a reference and a query chromosome and builds pairwise co-linear alignment chains; *RepeatFiller* [2] and *chainCleaner* [3] improve the pairwise alignment chains; *chainNet* [1] generates pairwise alignment nets by building a hierarchical collection of chains or parts of chains such that each locus in the reference is covered by at least one alignment to the query; *NetFilterNonNested* [3] removes low scoring and non-syntenic parts of nets in a non-nested fashion to generate the final filtered pairwise alignments. These 119 pairwise alignments are the input for *MultiZ* [4], which computes the multiple sequence alignment of 120 mammals.

The application of these tools does not require any manual manipulation. The only manual step was to construct the phylogenetic tree of these mammals from the literature.

I'd like to see the multiple sequence alignment of the 480 UCEs, however, the MAF files contained too many uninformative alignments. Most of the alignments are short and not conserved.

We agree with the reviewer that it is somewhat complicated to obtain the alignments of only UCE regions from the entire multiple whole genome alignment, which, of course, includes many less or not conserved regions. Therefore, we now provide the alignments of the UCEs in FASTA format at:

<https://bds.mpi-cbg.de/hillerlab/120MammalAlignment/Human120way/data/uce/>

As mentioned above, we now also setup a genome browser showing the 120-mammal alignment at <https://genome-public.pks.mpg.de/index.html>. Clicking on the “whole genome alignment of 120 mammals” link on that page brings the user to a genomic locus of one of the UCE and our alignment shows how similar the sequence of mammals is. In contrast, genomic loci up- and downstream of this UCE region are less well conserved.

Secondly, the authors claimed the number of species included in the genome alignment is a key factor of affecting the power of comparative analyses, thus the authors generated a genome alignment of 120 mammals. However, I was wondering how the number of species affect the power of comparative analyses. The authors should provide another analyze that includes fewer number of species. For example, the authors could generate another genome alignment of mammals with half of species in each mammalian order.

We thank the reviewer for this suggestion. To illustrate the impact of the number of species on comparative analysis, we have reduced our 120-mammal alignment to half the species and re-analyzed the lower and upper bound for the number of positions within UCEs showing variability. We have added to the main text:

“Considering only 60 instead of all 115 non-human placental mammals in this analysis, we obtained average upper and lower bound estimates of 11.8% (median 9.8%) and 2.7% (median 1.9%), respectively (Figure 3A), indicating that fewer species underestimate UCE variability.”

This analysis indeed shows that a reduced number of species underestimates UCE variability and demonstrates that the number of included species affects the power and accuracy of comparative analysis.

These new results have been integrated into panel A in Figure 3.

Thirdly, the authors identified huge number of conserved elements, however, the definition of conserved elements is not clear. The definition of an ultraconserved element is an interval of at least 200bp with 100% identity [45]. I was wondering what the difference of definitions between conserved elements and ultraconserved elements is. According to [18], conserved elements are defined as intervals of at least 100bp with >70% identity. However, the author used PhastCons to identify conserved regions with expected-length=45 and target-coverage=0.3. It seems a bit confusing about the definition of conserved elements.

We apologize that the different definitions of conserved elements were not clear in our previous manuscript, and we have accordingly improved the clarity of the manuscript at several places, as described below.

Reference 18 states in the introduction that “Most groups have used pairwise alignments and simple, percent identity-based methods for identifying conserved elements. For example, Dermitzakis et al. (2002) and Nobrega et al. (2003) have defined conserved elements as intervals of at least 100 bp with >70% identity.”. However, reference 18 (Siepel et al. 2005) developed an improved, now state-of-the-art method PhastCons that does not rely on fixed length and identity cut-offs. Instead, PhastCons is a two-state phylogenetic Hidden Markov Model (HMM) that infers genomic regions that are more likely to evolve under evolutionary constraint than evolving neutrally. To this end, PhastCons relies on a phylogenetic tree with branch lengths representing the number of substitutions per neutral site. The parameters expected-length=45 and target-coverage=0.3 are not used as fixed cut-offs, but instead are converted into the transition parameters of the two HMM states, as described in reference 18 on page 1047-1048.

We chose expected-length=45 and target-coverage=0.3, as these are the parameters were empirically determined by the UCSC genome browser group (and also our group) for mammalian genome alignments.

There is a key difference between a **conserved element**, as identified by PhastCons representing a widely-used method for this task, and the original definition of an **ultraconserved element**. An **ultraconserved element**, as defined in reference 45, is at least 200 bp long and identical between human, mouse and rat. It happens that these UCEs are in general also highly conserved among other mammals and other vertebrates, but this was not a requirement. A **conserved element** implies that PhastCons estimates a higher likelihood that the respective genomic region evolves under constraint. Thus, a conserved element does not have to be identical between human, mouse and rat, and it can be shorter than 200 bp. In general, ultraconserved elements are a small subset of conserved elements.

To clarify these points, we made several text changes:

“Second, in addition to annotating protein-coding genes, we annotated genomic regions that likely evolve under evolutionary constraint (purifying selection). To this end, we used PhastCons [18], a phylogenetic Hidden Markov Model method, and GERP++ [42], a method that directly measures the number of substitutions per site that were rejected by purifying selection. We applied both methods to detect regions constrained across all mammals in our alignment.”

“We focused on a subset of highly conserved elements, called ultraconserved elements (UCEs), that have attracted much attention as deletions of several of these elements does not affect cellular fitness and resulted in viable organisms [43-45]. UCEs were originally defined as genomic regions that are at least 200 bp long (the largest UCE is 779 bp long) and have identical sequences between human, mouse and rat [46]. Despite the fact that only three mammals were used to identify these genomic regions, UCEs are also highly conserved in other mammals and typically align to non-mammalian vertebrates [47].”

Fourthly, the author did not explain what an intact exon alignment is. It is estimated there were, on average, 5.48 exons per gene. How did the author define a human gene has intact exon alignments in mammals? If a human gene contains 10 exon and only one of its exon is aligned with chick gene, will it be counted or not?

Thank you for noticing that the definition of an intact exon alignment was missing. We have now added:

“Intact exons are defined as having an intact open reading frame without premature stop codons, and two consensus splice sites (internal exons) or one consensus splice site and a start (first exon) or stop (last codon) codon. “

We counted genes, where at least one of the human exons had an intact exon alignment to another mammal (we did not align the human genome to chicken), for the following reason that we added to the text:

“Since intact exons can be missing due to assembly gaps and assembly base errors [32, 34, 40, 41], we determined for each species the number of genes where at least one intact exon was annotated. We found that between 15,868 and 18,047 of the human genes have at least one intact exon alignment in placental mammals (Figure 1).”

Lastly, the authors should provide more comprehensive observations about the genome alignment of 120 mammals. For example, the average of sequence identity or the average number of conserved elements in each mammalian order.

As suggested, we have computed for each of the 119 non-human mammals how many bases align to the human genome and which fraction of the human genome this comprises. These statistics have been added to Supplementary Table 1.

We use all 120 mammals in our alignment to infer genomic regions under constraint with PhastCons or GERP++, as now clarified in the first Results paragraph:

“We applied both methods [PhastCons and GERP++] to detect regions constrained across all mammals in our alignment. PhastCons and GERP++ identified 13,257,408 and 1,612,714 conserved elements, respectively.”

Therefore, it is not possible to determine the average number of conserved elements per mammalian order, as by definition these conserved regions are under constraint across all analyzed mammals.

minor: Human UCE sequences are conserved in the chicken genome with an average of 95% identity rather than 96%[45].

Reference 45 writes: “Nearly all of these elements also exhibit extremely high levels of conservation with orthologous regions in the chicken genome [467 out of 481 (467/481) = 97% of the elements aligning at an average of **95.7% identity** …”

We used the rounded percentage (96%) in our manuscript.

**Reviewer #2:**

The authors present a valuable resource for comparative genomics analysis and demonstrate important insights into the sequences of ultraconserved elements and conserved enhancers.

My minor comments and suggestions are below:

The definition of conserved placental mammal specific enhancers is based on the presence of at least one shared 10-mer across all placental mammal clades, assumming that the 10-mer represents the typical size for transcription factor (TF) binding sites. It would be interesting to predict which TF binding sites are enriched in all of those 10-mers identified for 1820 enhancers and if these TFs are related to placenta-related genes. Results from this analysis would serve as orthogonal validation of the GREAT enrichment analysis.

We thank the reviewer for the suggestion. We would first like to mention that we also refined the set of placental mammal specific FANTOM enhancers, defined as enhancers have at least one conserved 10-mer in all major placental mammal clades but have no aligning sequence in marsupials and the platypus. While we previously used the score-filtered pairwise alignments that were used to generate the multiple genome alignment to exclude FANTOM enhancers that do align to marsupials or platypus, we have now use all unfiltered pairwise alignments for this filter step. As a result, we now used a slightly smaller (658 vs. 732) but more stringent set of placental mammal specific enhancers that show the same placenta-related enrichments as before. We have also used the latest GREAT webserver version 4 (available since August this year) to recompute the enrichments. The results are essentially the same.

As suggested, we have investigated which transcription factor binding motifs are enriched the conserved 10-mers in the 658 placental mammal specific enhancer regions, using the AME method from the MEME suite of tools. The enriched motifs are listed in new Supplementary Tables 8 and 9 and this new analysis revealed interesting new insights, as described in the text:

“Next, we investigated whether the set of conserved 10-mer sequences of the 1,820 mammal-conserved and 658 placental mammal-specific enhancers are enriched in transcription factor binding motifs. Using Analysis of Motif Enrichment (AME) from the MEME suite [60, 61], we found enrichments for motifs of several ETS (E26 transformation-specific) and AP-1 (activating protein-1) transcription factors in both 10-mer sets (Supplementary Tables 8 and 9). These transcription factors have various roles in development, cell proliferation and differentiation [62, 63]. In agreement with GO ‘artery morphogenesis’ (GO:0048844) and MGI ‘abnormal artery development’ (MP:0003410) gene enrichments of mammal-conserved enhancers (Supplementary Tables 3 and 4) and the GO ‘labyrinthine layer blood vessel development’ (GO:0060716) gene enrichment of placental mammal-specific enhancers (Supplementary Table 7), ETS family members FLI1, ERG and ETV2, whose motifs are enriched in the 10-mer sets, are involved in hematopoiesis and endothelial development [64, 65]. Interestingly, AP-1 family members JUN, JunB and FOS, whose motifs are also enriched in the 10-mer sets, are involved in trophoblast cell invasion into the uterus and essential for placentation [66, 67]. This agrees with placenta-related gene enrichments of placental mammal-specific enhancers (Supplementary Tables 6 and 7) and supports placenta-related functions of these enhancers. Furthermore, 10-mers in the placental mammal-specific enhancers exhibit enriched motifs for FOXP3 (forkhead-box-protein P3). This transcription factor has been linked to preeclampsia, a pregnancy related disorder characterized by high blood pressure [68, 69].”

Among the FANTOM conserved enhancers, are there enhancers with experimental validation to be active enhancers for human and mouse as reported in other databases such as the VISTA database?

We overlapped the FANTOM enhancers with conserved 10-mers with the human VISTA enhancers and found that only 8 placental-mammal specific and 63 of these elements overlap. This is probably not surprising as VISTA enhancers are only tested by transgenic reporter assays at mouse embryonic day 11.5 and the total number of human VISTA is enhancers is only 1912. In contrast, FANTOM enhancers are derived from measurements in most major human tissues and cell types.

We would also like to mention that while FANTOM enhancers in general have not been subjected to an independent enhancer assay (e.g. transgenic or luciferase assay), they are based on experimental data (bidirectional expression patterns of eRNAs that are a hallmark of enhancers). Furthermore, we limited our analysis to the conservative set of 38,548 FANTOM enhancers that are considered robust enhancers.

What improvements or new features does the Human120Way alignment have compared to other publicly available enhancers, for example in comparison with the multiple alignment of 144 vertebrate genomes, which were also contains the mapping of human genes to other 133 species using CESAR (Sharma and Hiller, 2017)?

The main difference between the 120-mammal (this manuscript) and the 144-vertebrate (2017 paper) alignment is that the new alignment focuses entirely on mammals and includes many more species (120 vs. 74), which facilitates new analyses such as computing a lower bound for UCE variability, as shown in our manuscript.

In addition, there are other advances. First, we used a new method RepeatFiller that improves the completeness of alignments between repetitive regions, making the 120-mammal alignment an improved resource to study co-option and evolution of transposons and other types of repeats. Second, we included 12 updated genome assemblies that have become available since and have a higher contiguity and completeness. Examples are chimp (panTro4 vs. panTro5), gorilla (gorGor3 vs. the new PacBio-based gorGor5), sheep (oviAri3 vs. oviAri4), horse (equCab2 vs. equCab3), or pig (susScr3 vs. susScr11). All species with updated assemblies or species newly added are now indicated in Supplementary Table 1 column G. Third, we used an updated human Ensembl build (version 96 vs. 78 that was used in the 2017 paper) to project genes to non-human mammals. The updated Ensembl version together with improved assemblies also results in improved gene projections. For example, while we previously annotated 158,024 non-overlapping exons for the pig genome assembly (susScr3), we now annotated 185,548 exons for the updated pig genome assembly (susScr11), which is a 17% increase in the number of annotated exons.

We added this information to the text:

“The main difference between this 120-mammal alignment and our previous 144-vertebrate alignment [16] is that the former focuses entirely on mammals and includes many new species (120 vs. 74 mammals, see Supplementary Table 1). In addition, we updated genome assemblies of twelve species that were already included in the previous alignment (species are marked in Supplementary Table 1). Finally, the 120-mammal alignment used RepeatFiller [83] to improve the completeness of alignments between repetitive regions.”

Regarding Supplementary Table S2, it appears the variability measured by lower bounds and the upper bounds is inversely proportional to the length, i.e. longer regions have lower values.

We thank the reviewer for this careful observation. We have now tested whether there is a correlation between UCE length and variability, and added to the main text:

“To investigate factors associated with UCE variability, we first tested whether there is a correlation between the percentage of variable columns and the length of UCEs. We found a weak but significant negative correlation (Kendall’s tau of -0.11 and -0.12 for the lower and upper bound variability, both p-values < 10-3; Supplementary Figure 2), indicating that longer UCEs tend to have a lower percentage of variable columns.”

The new Supplementary Figure 2 is:

**Supplementary Figure 2:** Relationship between the variability and length of UCEs.

Scatter plots show that there is a weak negative correlation between the fraction of variable columns and the length of UCEs.

(A) For the lower bound value for the fraction of variable columns (only considering shared substitutions), we obtain Kendall’s tau of -0.11 with p-value < 10^-3^.

(B) For the upper bound value for the fraction of variable columns (considering all substitutions), we obtain Kendall’s tau of -0.12 with p-value < 10^-3^.

This indicates that larger UCEs tend to be slightly less variable than smaller UCEs. Kendall’s tau is preferred over Spearman’s rank correlation if the data contains ties.

It will be useful to describe in more details, how the UCEs were determined and explain why their lengths are different. To make the bounds comparable between regions, the authors may consider weighting the variability by length for each region.

We changed the manuscript to clarify this. UCEs are obtained from UCbase2.0 (as described in the methods) and are identical to the UCEs originally defined in the Bejerano *et al.* 2004 reference. We added more text clarifications on how they were defined by Bejerano *et al.*:

“UCEs were originally defined as genomic regions that are at least 200 bp long (the largest UCE is 779 bp long) and have identical sequences between human, mouse and rat [46]. Despite the fact that only three mammals were used to identify these genomic regions, UCEs are also highly conserved in other mammals and typically align to non-mammalian vertebrates [47].”

We added the length of the largest UCE (779 bp) to indicate that many UCEs are longer than 200 bp.

We also make clear that UCEs represent a subset of conserved genomic regions:

“We focused on a subset of highly conserved elements, called ultraconserved elements (UCEs), …”

We would like to emphasize that we already normalized for differences in UCE length by creating 100 bins, when computing the relative distribution of variable columns within UCEs (Figure 3B). This is described in the methods:

“To investigate how variable positions are distributed within UCEs, we had to account for the different lengths of UCEs. To this end, we normalized the positions of each UCE into 100 equally sized bins.”

Very minor: Use a full name for CESAR (Coding exon-structure aware realigner) when it is first mentioned

Thank you. We now write: “We used the human gene annotation and Coding Exon-Structure Aware Realigner (CESAR) to provide comparative gene annotations for all 119 non-human mammals.”
